# Jack-of-all-trades effects drive biodiversity–ecosystem multifunctionality relationships in European forests

**DOI:** 10.1038/ncomms11109

**Published:** 2016-03-24

**Authors:** Fons van der Plas, Peter Manning, Eric Allan, Michael Scherer-Lorenzen, Kris Verheyen, Christian Wirth, Miguel A. Zavala, Andy Hector, Evy Ampoorter, Lander Baeten, Luc Barbaro, Jürgen Bauhus, Raquel Benavides, Adam Benneter, Felix Berthold, Damien Bonal, Olivier Bouriaud, Helge Bruelheide, Filippo Bussotti, Monique Carnol, Bastien Castagneyrol, Yohan Charbonnier, David Coomes, Andrea Coppi, Cristina C. Bastias, Seid Muhie Dawud, Hans De Wandeler, Timo Domisch, Leena Finér, Arthur Gessler, André Granier, Charlotte Grossiord, Virginie Guyot, Stephan Hättenschwiler, Hervé Jactel, Bogdan Jaroszewicz, François-Xavier Joly, Tommaso Jucker, Julia Koricheva, Harriet Milligan, Sandra Müller, Bart Muys, Diem Nguyen, Martina Pollastrini, Karsten Raulund-Rasmussen, Federico Selvi, Jan Stenlid, Fernando Valladares, Lars Vesterdal, Dawid Zielínski, Markus Fischer

**Affiliations:** 1 Institute of Plant Sciences, University of Bern, Altenbergrain 21, CH-3013 Bern, Switzerland; 2 Senckenberg Gesellschaft für Naturforschung, Biodiversity and Climate Research Centre, Senckenberganlage 25, 60325 Frankfurt, Germany; 3 Faculty of Biology, Geobotany, University of Freiburg, Schänzlestrasse 1, 79104 Freiburg, Germany; 4 Forest & Nature Lab, Department of Forest and Water Management, Ghent University, Coupure links 653, B-9000 Ghent, Belgium; 5 Systematic Botany and Functional Biodiversity, University of Leipzig, Johannisallee 21, 04103 Leipzig, Germany; 6 German Centre for Integrative Biodiversity Research (iDiv) Halle-Jena-Leipzig, Deutscher Platz 5e, 04103 Leipzig, Germany; 7 Forest Ecology and Restoration Group, Department of Life Sciences, University of Alcalá, Alcalá de Henares, 28805 Madrid, Spain; 8 Department of Plant Sciences, University of Oxford, S Parks Road, Oxford OX1 3RB, UK; 9 Terrestrial Ecology Unit, Department of Biology, Ghent University, K. L. Ledeganckstraat 35, B-9000 Ghent, Belgium; 10 INRA, UMR 1202 BIOGECO, F-33610 Cestas, France; 11 University Bordeaux, BIOGECO, UMR 1202, F-33600 Pessac, France; 12 Faculty of Environment and Natural Resources, Chair of Silviculture, University of Freiburg, Fahnenbergplatz, 79085 Freiburg, Germany; 13 Institute of Biology/Geobotany and Botanical Garden, Martin Luther University Halle Wittenberg, Am Kirchtor 1, 06108 Halle, Germany; 14 INRA, UMR EEF, Allée de l'Arboretum, 54280 Champenoux, France; 15 Faculty of Forestry, Stefan cel Mare University of Suceava, Universitatii Street 13, Suceava 720229, Romania; 16 Department of Agri-Food Production and Environmental Science (DISPAA), Lab. of Applied and Environmental Botany, University of Firenze, Via G. La Pira 4, 50121 Firenza, Italy; 17 Laboratory of Plant and Microbial Ecology, University of Liège, Botany B22, Chemin de la Vallée 4, 4000 Liège, Belgium; 18 Forest Ecology and Conservation, Department of Plant Sciences, University of Cambridge, Downing Street, Cambridge CB2 3EA, UK; 19 Department of Biogeography and Global Change, National Museum of Natural Sciences, MNCN, CSIC, Serrano 115 bis, 28006 Madrid, Spain; 20 Department of Geosciences and Natural Resource Management, University of Copenhagen, Rolighedsvej 23, 1958 Frederiksberg C, Denmark; 21 Division of Forest, Nature and Landscape, University of Leuven, Celestijnenlaan 200E, B-3001 Leuven-Heverlee, Belgium; 22 Natural Resources Institute Finland, PO Box 68, FI-80101 Joensuu, Finland; 23 Swiss Federal Research Institute WSL, Zürcherstrasse 111, CH-8903 Birmensdorf, Switzerland; 24 Earth and Environmental Sciences Division, Los Alamos National Laboratory, PO Box 1663, Los Alamos, New Mexico 87545, USA; 25 INRA, UMR 1201, DYNAFOR, F-31326 Castanet-Tolosan, France; 26 Centre of Evolutionary and Functional Ecology (CEFE UMR 5175 – University of Montpellier – University Paul-Valéry Montpellier – EPHE), 1919 route de Mende, 34293 Montpellier, France; 27 Białowieża Geobotanical Station, Faculty of Biology, University of Warsaw, Ilji Miecznikowa 1, 02-096 Warsaw, Poland; 28 School of Biological Sciences, Royal Holloway University of London, Egham, Surrey TW20 0EX, UK; 29 Department of Forest Mycology and Plant Pathology, Swedish University of Agricultural Sciences, PO Box 7026, SE-750 07 Uppsala, Sweden; 30 Departamento de Biología y Geología, ESCET, Universidad Rey Juan Carlos, c/ Tulipan s.n., 28933 Móstoles, Spain

## Abstract

There is considerable evidence that biodiversity promotes multiple ecosystem functions (multifunctionality), thus ensuring the delivery of ecosystem services important for human well-being. However, the mechanisms underlying this relationship are poorly understood, especially in natural ecosystems. We develop a novel approach to partition biodiversity effects on multifunctionality into three mechanisms and apply this to European forest data. We show that throughout Europe, tree diversity is positively related with multifunctionality when moderate levels of functioning are required, but negatively when very high function levels are desired. For two well-known mechanisms, ‘complementarity' and ‘selection', we detect only minor effects on multifunctionality. Instead a third, so far overlooked mechanism, the ‘jack-of-all-trades' effect, caused by the averaging of individual species effects on function, drives observed patterns. Simulations demonstrate that jack-of-all-trades effects occur whenever species effects on different functions are not perfectly correlated, meaning they may contribute to diversity–multifunctionality relationships in many of the world's ecosystems.

## Abstract (web summary)

How biodiversity is linked to multiple ecosystem functions is not fully understood. Here, the authors show that a new mechanism, which they term the 'jack-of-all-trades' effect, best explains patterns of tree diversity and ecosystem multifunctionality in European forests.

## Graphical abstract

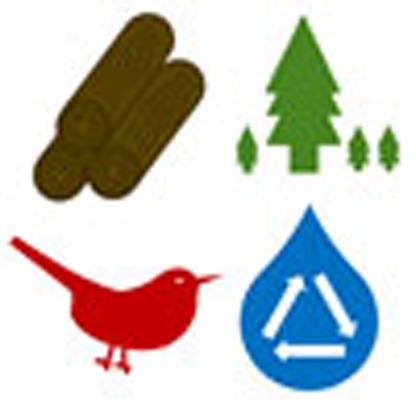

There is considerable evidence that communities with high biodiversity are better able to deliver ecosystem functions and services than species-poor communities^1^,^2^,^3^,^4^ and that this relationship is even stronger when multiple functions are considered (multifunctionality^5^,^6^,^7^,^8^,^9^,^10^,^11^,^12^,^13^,^14^,^15^). For individual ecosystem functions, two mechanisms have been identified by which biodiversity can promote them: (i) increased functioning due to resource partitioning or facilitation (‘complementarity'), and (ii) the greater likelihood of diverse communities being dominated by a competitively superior, high-performing species (‘selection')^16^,^17^,^18^. However, it remains an open question whether these or other mechanisms also drive the relationship between diversity and ecosystem multifunctionality.

Multifunctionality measures are calculated from the values of multiple individual functions^5^,^6^,^7^,^8^,^9^,^10^,^11^,^12^,^13^,^14^,^15^. It therefore follows that the mechanisms that promote levels of individual functions in diverse communities (complementarity and selection) should also underlie relationships between biodiversity and ecosystem multifunctionality. However, to date this has never been quantified due to a lack of analytical tools. Additionally, we propose that a third and not mutually exclusive mechanism, which we term ‘the jack-of-all-trades effect', may underlie diversity–multifunctionality relationships. This mechanism gets its name from an English language saying ‘Jack-of-all-trades, master-of-none' in which Jack is a tradesman who can perform many trades to an adequate level, but is not extremely highly skilled in any. Ecologically, this mechanism relies upon the assumptions that different species promote different functions and that species effects on ecosystem functioning are proportional to their abundance in a community^19^,^20^. In such cases, the ecosystem functioning of a multispecies mixture will equal the biomass-weighted average of the function levels of monocultures of its component species (Fig. 1; Supplementary Fig. 1). As a result, in the absence of other biodiversity effects, such as complementarity or selection, function levels in diverse communities are expected to be intermediate and never as extremely low or high as in some monocultures (Fig. 1). In other words, a diverse community (‘Jack') would have moderate levels of most or all ecosystem functions (‘trades') but this averaging would also prevent it from having the highest possible levels of any function, making it a ‘master-of-none'. Hence, this jack-of-all-trades effect might underlie both positive and negative relationships between biodiversity and multifunctionality, when it is defined as the number of functions performing above certain threshold levels (as done in refs 7, 12, 15). This proposed jack-of-all-trades mechanism is broadly analogous to the averaging effects underlying the ‘portfolio effects' that drive both higher stability of financial assets when partitioned across multiple stocks^21^ and higher temporal stability of biomass production in diverse communities^22^,^23^. In all these cases, a high diversity decreases variability, either across multiple functions (jack-of-all-trades effects) or across time^21^,^22^,^23^, through averaging mechanisms.

In this study we used a novel approach that does not require data on species-specific contributions to plot-level functioning, to quantify the relative importance of selection, complementarity and jack-of-all-trades mechanisms in driving biodiversity effects on forest multifunctionality. The first step in doing this was to elucidate the observed relationships between tree diversity and both individual functions and multifunctionality in European forests. Forests play a vital role in delivering numerous ecosystem services, including timber provision, recreation and the regulation of pests, floods, water quality and climate^11^,^24^,^25^. Data were collected in 209 mature forest plots located in six European countries (Spain, Italy, Romania, Poland, Germany and Finland), representing all major European forest types^26^. These contained 15 regionally dominant tree species. As the study aimed to quantify biodiversity effects on ecosystem functioning, plots were selected to differ as much as possible in tree diversity, while minimizing any (co-)variation in other drivers of ecosystem function, including altitude, soil texture, soil pH and species composition and evenness^26^. Hence, although observational, our study aimed to mimic the design of biodiversity experiments by selecting monocultures of all species present in mixed cultures and by having a balanced number of compositions at each level of species richness. However, in contrast to many synthetic community assemblages in experiments, the age structure of the communities is not uniform and the assembly history is unknown. In each plot, tree species richness and 16 ecosystem functions or properties (‘functions' hereafter), including timber production, decomposition rates, drought and pest resistance and bird diversity, were measured. We quantified multifunctionality as the number of ecosystem functions in a plot with levels exceeding a certain threshold^7^, expressed as a percentage of observed maximum functioning across all plots from the same country. Because the relationship between biodiversity and multifunctionality can depend on the level of functioning required^12^,^15^, we calculated 99 multifunctionality variables, covering threshold values ranging from 1 to 99% (ref. 12) and using a novel partitioning approach, we quantified the contributions of different mechanisms to biodiversity–multifunctionality relationships. Our results showed that jack-of-all-trades effects cause positive relationships between tree diversity and multifunctionalty when low levels of functioning are required, but negative relationships when very high levels of functioning are desired. Simulation analyses show that in the absence of effects of complementarity or selection, such patterns are always to be expected as long as different species support different functions, meaning that, in addition to other well-known mechanisms, jack-of-all-trades effects may drive relationships between biodiversity and multifunctionality in many of the world's ecosystems.

## Results

### Observed tree diversity–multifunctionality relationships

Using mixed effects models, in which we tested for potentially confounding covariates including species evenness, soil pH, community composition, proportion of coniferous trees, altitude and country (the latter capturing the effects of numerous covariates such as temperature, rainfall and the regional species pool) and retained those factors with significant effects (that is, proportion of coniferous trees, altitude, community composition and country), we found that (i) only 2 out of 16 individual ecosystem functions were significantly related to diversity and hence (ii) the average relationship between individual ecosystem functions and tree diversity was non-significant (Fig. 2b). In contrast, we found strong relationships between multifunctionality and tree diversity. At low to intermediate thresholds (1–45%), tree diversity was significantly positively related to ecosystem multifunctionality (Fig. 2a; Supplementary Table 1), a finding that is consistent with studies performed in grasslands and freshwater systems^6^,^7^,^8^,^12^,^14^,^15^. Positive relationships with biodiversity peaked at the 37% threshold, where each additional tree species was associated with 0.52 extra ecosystem functions (Fig. 2c; Supplementary Table 1). This relationship was reversed and became negative at much higher (76–99%) thresholds (Fig. 2d; Supplementary Table 1). For example, at a 90% performance threshold, an increase from one to five tree species was associated with a loss of 1.5 ecosystem functions. Hence our results show a pattern where diverse tree communities can provide almost all functions at intermediate levels (Fig. 2c), but only one at high levels (Fig. 2d). In contrast, species-poor forests provide fewer functions above intermediate thresholds, but relatively more at high thresholds, in line with the jack-of-all-trades hypothesis (Fig. 2c,d). Additional analyses demonstrated that these diversity–multifunctionality relationships were insensitive to the identity of the functions included (Supplementary Fig. 2; Supplementary Table 2), independent of covariates (Supplementary Fig. 3) and consistently linear (Supplementary Fig. 3), across different countries and forest types (Supplementary Table 1; Supplementary Fig. 4). We therefore provide strong evidence that biodiversity affects forest multifunctionality more strongly than it does individual ecosystem functions.

### Effects of complementarity

**Figure 1:**
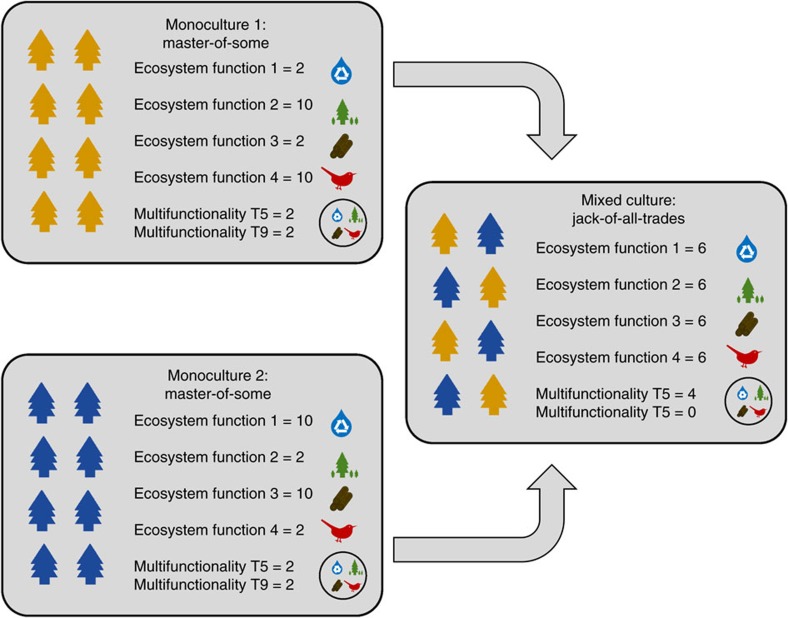
Hypothetical example where the mixing of two species causes a ‘jack-of-all-trades, but master-of-none' effect. The two monocultures (left panels) each support two functions at high levels and two functions at low levels. In the absence of complementarity or selection, the mixing of the two species results in a combined functioning that is intermediate between monoculture function values of the component species. As a result, when multifunctionality is quantified as the number of functions exceeding a moderate threshold value (for example, a value of 5, as indicated by multifunctionality T5), a positive diversity–multifunctionality relationship is found, while this relationship is negative at a higher threshold value of 9.

**Figure 2:**
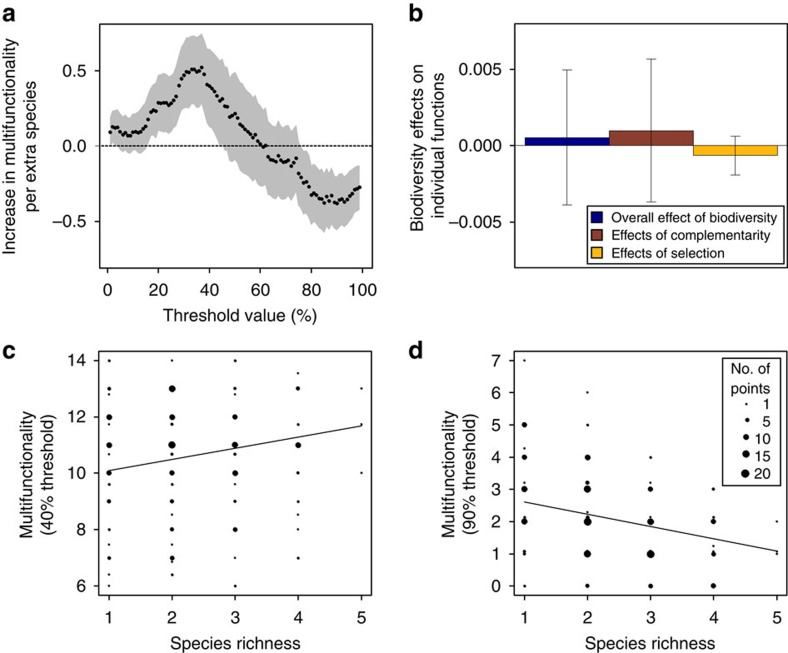
The effects of tree biodiversity on observed ecosystem multifunctionality and individual ecosystem functions. Based on linear mixed models (*N*=209 plots). (**a**) The biodiversity effect (increase in number of functions above a threshold level per extra species) for a range of multifunctionality thresholds. The dotted, horizontal line indicates a biodiversity effect of zero. The grey polygon indicates the 95% confidence interval. (**b**) Average (across functions) overall effects of diversity, and effects of complementarity and selection (±s.e.m.) on individual ecosystem functions are non-significant (all *P*>0.05). (**c**,**d**) The multifunctionality value (number of functions above a 40% (**c**) or 90% (**d**) threshold value) as a response to species richness (both *P*<0.05).

**Figure 3:**
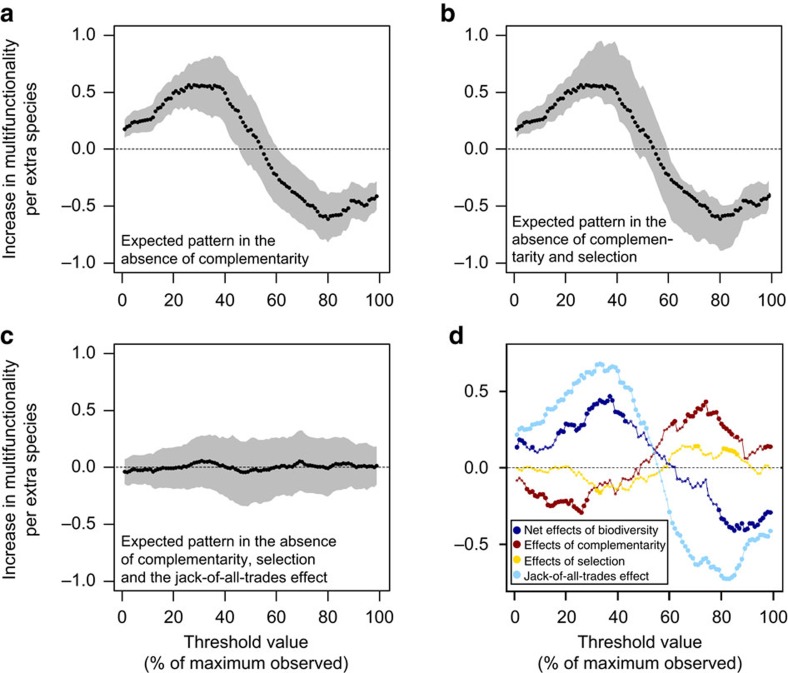
The biodiversity effect on multifunctionality partitioned into different mechanisms. Based on linear mixed models (*N*=209 plots). The expected biodiversity effect is shown for a scenario where (**a**) complementarity (MF_exp1_), (**b**) both complementarity and selection (MF_exp2_) and (**c**) complementarity, selection and the jack-of-all-trades effects (MF_exp3_) are excluded, for a range of multifunctionality threshold values. (**d**) The net biodiversity effect on multifunctionality (blue line), partitioned into complementarity (red), selection (yellow) and the jack-of-all-trades (green) effects. The dotted, horizontal lines show a biodiversity effect of zero. The grey polygon represents the 95% confidence area in **a**–**c**, while points significantly deviating from zero are extra-large in **d**.

As a next step we investigated why diversity–multifunctionality relationships were stronger than those between biodiversity and individual ecosystem functions. We did this by developing a new analytical approach that estimates the effects of complementarity, selection and jack-of-all-trades mechanisms on both individual ecosystem functions and ecosystem multifunctionality. This was achieved by using monoculture data to simulate artificial communities in which the three biodiversity–multifunctionality mechanisms were sequentially removed, thus eliminating their effect. We first investigated, for each threshold, the extent to which biodiversity affects multifunctionality in the absence of complementarity. This was done by calculating expected function values for mixed communities based on biomass-weighted function values obtained from monoculture plots, thus eliminating non-additive diversity effects. Any deviation between observed functioning and these expected function values, where non-additive effects were excluded, were then attributed to complementarity effects (Methods, equation (4)). In this simulation biodiversity–multifunctionality relationships were remarkably similar to observed patterns (Fig. 3a): biodiversity promoted multifunctionality at low to intermediate levels of functioning, but decreased it at higher levels of functioning. However, in this complementarity-free simulation, diversity decreased multifunctionality over an even larger range of threshold values (61–99%; Fig. 3a) than it does in real forests (Fig. 2a), thus demonstrating that the ‘master-of-none' negative biodiversity effect at high thresholds (Fig. 1), is ameliorated by positive complementarity (Fig. 3d). These weakly positive effects of complementarity on multifunctionality were not surprising, given that effects of complementarity on individual functions were, on average, also weakly positive (Fig. 2b).

### Effects of selection

**Figure 4:**
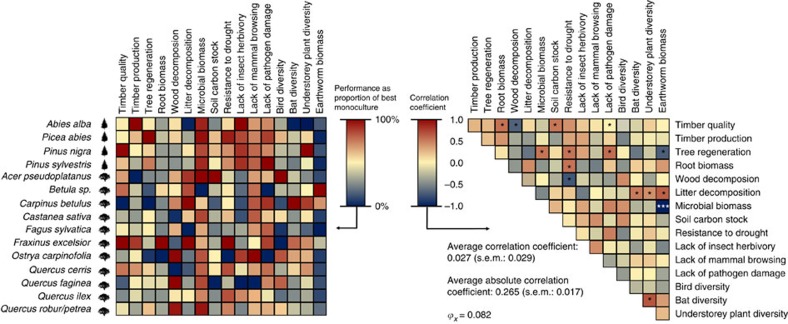
Tree species differ in their monoculture values of ecosystem functions. Left: scaled average ecosystem function values for each monoculture, after correcting for country differences in functions. Values indicate the proportion of the maximum value observed in any monoculture. Correcting for country differences in functions was done by calculating residuals (average species function value−average country function value). Right: species-level correlation coefficients between ecosystem functions, after correcting for country differences in functions. Negative correlations are shown in blue, positive ones in red. Significance correlations: *P<0.05; **P<0.01; ***P<0.001. *ϕ*_*x*_, is a metric describing the strength of matrix relationships^47^, that takes asymmetry into account (for example, with >2 variable, an average correlation coefficient of −1 is impossible) and is standardized between 0 (most negative relationships possible) and 1 (all correlations equal 1). The value calculated confirms that relationships between species effects on ecosystem functions are generally weak.

Next, we investigated how tree diversity would affect multifunctionality if, in addition to complementarity, selection was also excluded. In the absence of selection, relative biomass of individual species in mixtures is unrelated to the functioning of their monocultures^16^,^17^,^18^. Following this, we calculated expected functioning as before, but using equal biomass for all species present instead of actual species biomass. Removing effects of selection made little difference to expected ecosystem functioning (Fig. 3a versus Fig. 3b), indicating that selection had little impact on the tree diversity–multifunctionality relationship (Fig. 3d). Selection mechanisms on individual functions showed similar patterns: effects were weak and non-significant (Fig. 2b). However, note that selection may have been underestimated due to our study design, which only included plots with a high evenness in abundance^26^ and in which earlier competitive exclusion remains undetected. It is also important to note that since our statistical approach did not consider effects of tree diversity on total biomass when quantifying effects of complementarity, complementarity effects that operate through biomass overyielding, for example, via the provision of additional food for herbivores, might have been overlooked. Overyielding of biomass production has been as demonstrated in our study area^27^. However, as ‘net effects' (that is, effects of complementarity + selection) of biodiversity were calculated in a manner similar to previous approaches^18^, any underestimation of complementarity would be transferred to selection effects. Given that selection effects were generally weak (Fig. 3d) and most functions were not strongly related to annual biomass production (max *r*^*2*^=0.18; Fig. 4; Supplementary Table 3), such biases were probably minor in our study. Nevertheless, in cases where many ecosystem functions are strongly related to biomass production, care should be taken when using our analytical approach. Future studies, in which tree communities are monitored from establishment and for longer periods, could investigate whether the effects of complementarity and selection on diversity–multifunctionality relationships are stronger than those found here.

### Jack-of-all-trades effects

Finally, to remove jack-of-all-trades effects, we calculated multifunctionality using function values from a randomly selected monoculture from one of the observed component species. This removes the effect of averaging the extreme function values of monocultures that may occur in species mixtures (Fig. 1). As a result of this, replacing observed function values by randomly selected monoculture values caused the diversity–multifunctionality relationship to disappear (Fig. 3c), thus suggesting that the jack-of-all-trades effect is an important mechanism underlying the diversity–multifunctionality relationship in European forests. Correspondingly, jack-of-all-trades effects were almost identical to the overall effect of diversity on multifunctionality (Fig. 3d), with significantly positive effects at low (1–51%), but negative effects at high (61–99%) thresholds. Additional analyses showed that positive jack-of-all-trades effects at moderate threshold levels were consistent across countries (Supplementary Table 1; Supplementary Fig. 5), while effects of selection and complementarity were strong in some individual countries, but overall less strong than jack-of-all-trades effects due to a less consistent presence. Hence our results demonstrate an important role for the jack-of-all-trades effect, which causes diverse tree communities to be jacks-of-nearly-all trades (functions) (Fig. 2c), but a master of only one (Fig. 2d). In contrast, this mechanism makes species-poor forests jacks-of-many trades, but a master of a few (Fig. 2c,d).

### Jack-of-all-trades effects in theoretical communities

**Figure 5:**
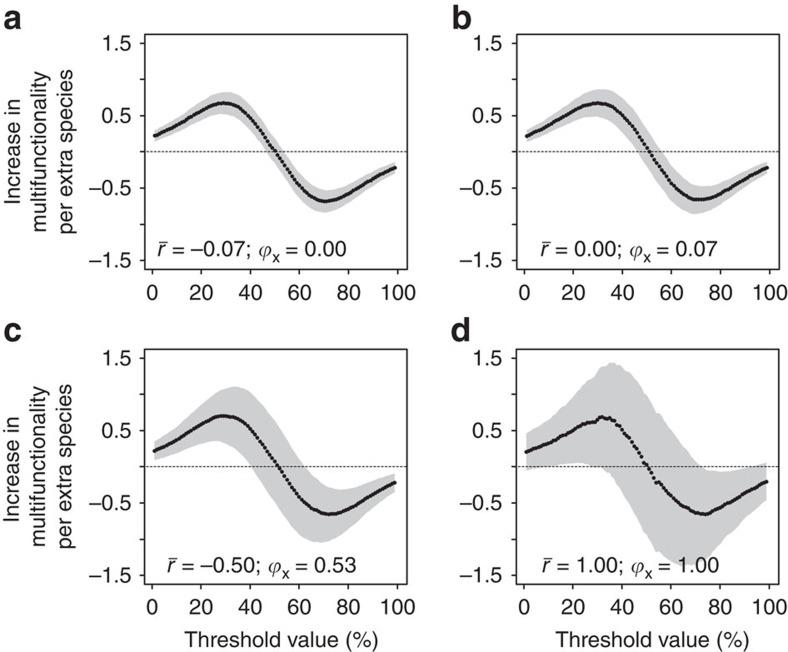
The biodiversity effect across a range of multifunctionality threshold values in theoretical communities. Artificial communities were created by randomly drawing species from an artificial, regional species pool. Average correlation coefficients between ecosystem function values of these monocultures are −0.07 (**a**), 0.00 (**b**), 0.50 (**c**) and 1.00 (**d**), while *ϕ*_*x*_ values^47^, which indicate overall correlation strength, range from 0 (indicating lowest possible average correlation coefficients) to 1 (maximally positive correlations, equivalent to a single-function scenario). Linear models (*N*=100) were used to quantify the biodiversity effect and the 95% confidence interval (grey polygon). The observed average correlation value among functions in monocultures in European forests was 0.027 (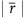
=0.265; Fig. 1). The dotted, horizontal line shows the *x* axis, where the biodiversity effect is zero.

Finally, we investigated whether jack-of-all-trades effects could be a general ecological phenomenon, by performing simulations where we created theoretical communities and varied the strength of correlation between the effects of species on different ecosystem functions. By randomly assembling communities, we created null expectations for scenarios where complementarity and selection are absent. Previous studies have shown that trade-offs in the effects of species on different functions can constrain the maximum level of multifunctionality that is achievable^8^. Our simulations extend these findings by showing that the ‘jack-of-all-trades, but master-of-none' relationship (Fig. 2a) can be expected as long as species effects on different ecosystem functions are not strongly, positively correlated (Fig. 5; Supplementary Table 4). Only when ecosystem functions are perfectly, positively correlated (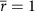), a case that is equivalent to studying a single function, does the jack-of-all-trades relationship became so inconsistent (that is, with very large confidence intervals) that it is rarely significant. In the European forests we studied, correlation coefficients between ecosystem functions among monocultures were weak (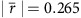; Fig. 4; Supplementary Table 3), thus explaining the strong jack-off-all-trades patterns.

## Discussion

While it is now widely established that biodiversity can promote the delivery of multiple ecosystem functions simultaneously^15^, no previous study has quantified the contribution of different underlying mechanisms to this relationship. Doing so is interesting because previous studies have demonstrated that relationships between biodiversity and ecosystem multifunctionality are often stronger than relationships between biodiversity and individual ecosystem functions^6^,^7^. This suggests that in addition to the well-known mechanisms that promote individual ecosystem functions in diverse communities, other mechanisms that have not been previously quantified, might boost multifunctionality in diverse communities even further. Our study confirmed this idea, by showing that in European forests, jack-of-all-trades effects caused communities with many tree species to have higher multifunctionality when moderate levels of functioning are required, but lower multifunctionality when very high levels of multifunctionality are desired. In addition, our simulation study showed that this mechanism could potentially be general and therefore contribute, together with other mechanisms, to relationships between biodiversity and multifunctionality in many of the world's ecosystems.

Averaging effects similar to the jack-of-all-trades mechanisms we described here have been previously proposed to affect relationships between biodiversity and individual functions^28^, although the likely inconsistency in the strength of these effects, compared with other diversity effects, on individual functions may have precluded further attention. Our study confirms these earlier findings, by demonstrating that when species effects on different ecosystem functions are maximally positively correlated (a case that is mathematically equivalent to a single-function scenario, as different functions are substitutable), then jack-of-all-trades effects are generally too inconsistent to be statistically detectable. However, our simulations also showed that when species effects on different ecosystem functions are weakly positively correlated or even negatively correlated, jack-of-all-trades effects are more consistent and statistically detectable over a large range of thresholds, as our data on European forest plots confirmed. These weakly positive or even negative correlations between ecosystem functions are to be expected as fundamental trade-offs in life history strategies, for example, the ‘fast-slow' spectrum of plants^29^, mean that a species' capacity to support some functions at high levels will compromise its ability to support others. For example, a slow growing tree is likely to produce high-quality timber and slowly decomposing litter, although it has a low rate of biomass accumulation. We therefore hypothesize that jack-of-all-trades effects, while forming a statistical null expectation, also have a biological basis and their strength, that, like other diversity effects, is likely to be modified by the degree of life history strategy variation within the species pool. Accordingly, the ‘jack-of-all-trades effect' can be viewed as a formalization of the notion that functional differences between species are the reason that diversity–multifunctionality relationships are even stronger than relationships between diversity and single ecosystem functions^6^,^7^,^9^,^11^,^12^,^14^. These ideas also have empirical support; a range of studies suggest that low correlations among species effects on different ecosystem functions are common place in a wide range of terrestrial and aquatic ecosystems, and among taxa ranging from plants to bacteria^7^,^8^,^30^, thus indicating the potential generality of jack-of-all-trades effects in driving biodiversity–multifunctionality relationships. In line with these patterns, clear ‘jack-of-all-trades-but-master-of-none' patterns have been observed in some studies^12^,^14^,^15^, while in others effects of biodiversity on multifunctionality were positive over an even larger range of threshold values^12^,^13^,^14^,^15^ and the ‘master-of-none' downturn was only observed at extremely high thresholds. While this does not imply an absence of jack-of-all-trades effects in these studies, it does indicate that complementarity and/or selection mechanisms can be strong enough to overcome the negative averaging effects seen at high thresholds.

Our finding that (i) the so far overlooked jack-of-all-trades effect is an important and widespread mechanism driving biodiversity–multifunctionality relationships in European forests and that (ii) this mechanism is likely to occur whenever species differ in their functional effects, is not only of fundamental importance, but could also have important implications for ecosystem management. Whenever species effects on individual ecosystem functions are not strongly positively correlated, positive effects of biodiversity on multifunctionality can be expected as long as only moderate levels of function are desired, even in the absence of other diversity-function mechanisms. Our simulations demonstrate that jack-of-all-trades effects are possibly a near universal mechanism throughout the world's ecosystems. In turn this indicates that conserving or promoting biodiversity in almost any ecosystem should ensure at least moderate levels of ecosystem multifunctionality. Finally, we should stress that jack-of-all-trades effects are not mutually exclusive to other biodiversity mechanisms. This was illustrated in our own system, where positive complementarity avoided significantly negative diversity–multifunctionality relationships over a large range (61–75%) of high threshold values. While the detected effects of complementarity and selection were relatively modest in our system, they have been found to be strong for some individual functions in our system^27^ (but see refs 31, 32 which describe inconsistent or neutral effects) and in many other systems^4^, where data on historical community compositions may have improved their detectability. Hence, in many systems, complementarity and selection may extend positive biodiversity effects towards even higher thresholds.

## Methods

### Plot selection and community characterization

In total, 209 mature forest plots measuring 30 × 30 m were used for this study, which is part of the FunDivEurope^26^ project. These plots were primarily established to investigate the role of the richness of regionally common and economically important ‘target' species on ecosystem functioning^26^ and were hence selected to differ as much as possible in the richness of these. In total, there were 15 target species (Fig. 4) across all 209 plots and between 1 and 5 abundant target species within plots. Target species contributed to >90% of the tree biomass in the plots and therefore we expected them to be most important for ecosystem functioning. Plots were distributed over six European countries: Finland, Germany, Italy, Poland, Romania and Spain (Supplementary Fig. 6), thereby covering six main European forest regions^26^. Hence, per region, there were ∼40 plots, as power analyses indicated that this should be sufficient to statistically detect existing diversity–functioning relationships^26^. Plots were carefully selected so that correlations between tree species richness and community composition, topography and potentially confounding soil factors were minimized^26^, thus ensuring robust tests of diversity–multifunctionality relationships. Most forest plots were historically used for timber production but are now managed by low frequency thinning or with minimal intervention^26^. Hence, species compositions and diversity patterns in forests are predominantly management driven and/or are the result of random species assembly, from the regional species pool. This design reduces the risk that other factors confound biodiversity effects on multifunctionality. All sites are considered as mature forests.

At each richness level, each target tree species was present in at least one plot, allowing us to statistically test for compositional effects (presence/absence of species) on multifunctionality. Since species evenness might also affect ecosystem functioning^33^, all plots were selected to have target species with similar abundances (with Pielou's evenness values above 0.6 in >91% of the plots). To reach this goal, we *a priori*^26^ decided to exclude locally rare target species (<2 individuals per plot) in richness measures.

To describe community composition and to estimate biomass values of each tree in each plot, we identified all stems ≥7.5 cm in diameter to species and permanently marked them (12,939 stems in total). For each stem we recorded diameter (to the nearest 0.1 cm, using diameter tape) and height (to the nearest 0.1 m, using a vertex hypsometer, Häglof AB, Sweden). In addition, we estimated the crown illumination index (CI) to characterize the dominance of each crown^34^. Diameter, height and CI measurements were used to estimate the aboveground biomass of each tree based on published biomass functions (see below). All selected equations were species specific, and whenever possible we chose functions developed for trees growing in similar forest types to those found at our sites. Plot-level biomass estimates were obtained by summing the biomass of all standing trees within a plot. Based on the biomass and abundance estimates, we found that non-target tree species on average contributed to only 6.25% of the individuals and 8.61% of the tree basal area in a plot. In monoculture plots, the target species contributed to 88.6% of the individuals and 95.3% of the tree basal area in a plot.

### Measurement of covariates

In each plot, altitude was recorded during plot selection. In addition, between May and October 2012, in each plot forest floor soil pH was determined in a solution of 0.01 M CaCl_2_ at a ratio of 1:10 for the forest floor material and 1:2.5 for the mineral soil. The solution was shaken for 2 h and the pH values were read with a pH metre (827 pH labs Metrohm AG, Herisau, Switzerland).

### Overview of ecosystem functions and properties

In each plot, 16 ecosystem functions and properties (‘ecosystem functions' or EFs hereafter), which we used to calculate multifunctionality, were measured. The measured EFs were wood quality, timber production, tree regeneration, root biomass, wood decomposition, litter decomposition, microbial biomass, soil carbon stock, resistance to drought, resistance to insect herbivory, resistance to mammal browsing, resistance to pathogen damage, bird diversity, bat diversity, understory plant diversity and earthworm biomass. All measured EFs have established links to supporting, provisioning, regulating or cultural ecosystem services.

### Timber quality

For timber quality measurements, in each plot dendrometric data and externally visible stem characteristics were recorded. The silvicultural quality assessment was based on stem characteristics that can be measured and evaluated non-destructively and rapidly along with a measurement of potentially influencing factors at the tree- and stand-level. For each tree within a plot, total height, height of the crown base, height of the lowest dead branch (>1 cm diameter) and type of fork (or steeply angled branch) were measured. In addition, the presence of the following stem quality parameters was recorded: curving, stem lean, epicormic branching, coppicing, pathogenic and other defects. Due to the multiple factors constituting stem quality and wood quality, a four-class stem quality grading scheme was used to aggregate all stem quality parameters collected for each tree into an appropriate stem quality score, allowing for the analysis of a single response variable across all regions, species diversity levels and compositions (see Supplementary Table 5 for details). The assessment of stem quality parameters was limited to the butt log of the tree, which represented the lowest 5 m of the stem for broadleaved tree species and a maximum of 10 m from the stem base for conifers. Multiples of the 5-m section were only considered if the second log showed at least quality class C=2, but only if the green crown base was above the section considered. It has been estimated that for most commercial species in Europe, these butt logs comprise up to 50–70% (softwood) and 80–95% (hardwood) of the total commercial tree value^35^. Plot-level timber quality was then calculated as the average timber quality of all the individual trees.

### Timber biomass and production

#### Wood cores

Tree ring data were used to reconstruct the past annually resolved wood production. Between March and October 2012, bark-to-pith increment cores (5 mm in diameter) were collected for a subset of trees in each plot following a size-stratified random sampling approach^27^,^36^. We cored 12 trees per plot in monocultures and 6 trees per species in mixtures (except in Poland, where only 5 cores per species were taken in all plots due to restrictions imposed by park authorities), for a total of 3,138 cored trees. Short of coring all trees within a stand, this approach has been shown to provide the most reliable estimates of plot-level productivity when using tree ring data, as it ensured that the size distribution of each plot is adequately represented by the subsample. Wood cores were stored in polycarbonate sheeting and allowed to air dry before being mounted on wooden boards and sanded with progressively finer grit sizes. A high resolution flatbed scanner (2,400 d.p.i. optical resolution) was then used to image the cores.

#### From tree rings to aboveground wood production

We followed a four step approach (i–iv) to estimate temporal trends in aboveground wood production (AWP, in MgC ha^−1^ per year) from tree ring data^27^,^36^. All analyses were performed in R (3.0.1; R Development Core Team 2013).

(i) Measuring growth increments from wood cores: we measured yearly radial growth increments (mm per year) for each cored tree from the scanned images. To minimize measurement errors associated with incorrectly placed ring boundaries, we crossdated each sample against a species-level reference curve obtained by averaging all ring-width chronologies belonging to a given species from a given site. In this process, 188 cores which showed poor agreement with reference curves were excluded from further analysis, giving a final total of 2,950 tree ring chronologies. Both radial growth measurements and crossdating were performed using CDendro (Cybis Elektronik & Data, Saltsjöbaden, Sweden). For the purposes of this study we limited our analysis to the 5-year period between 2007 and 2011.

(ii) Converting diameter increments into biomass growth: we combined radial increments and allometric functions to express the growth rate of individual trees in units of biomass. We calculated the average yearly biomass growth between 2007 and 2011 (*G*, kgC per year) of cored trees as *G*=(AGB_*t*2_−AGB_*t*1_) Δ*t*^−1^, where AGB_*t*2_ is the tree's biomass (estimated with equations presented in Jucker *et al*.^27^) in the most recent time period (that is, end of 2011) and AGB_*t*1_ is its biomass (estimated with equations presented in Jucker *et al*.^27^) at the previous time step (that is, end of 2006) Δ*t* and is the elapsed time (that is, 5 years). AGB_*t*1_ was estimated by replacing current diameter and height measurements used to fit biomass equations with past values. Past diameters were reconstructed directly from wood core samples by progressively subtracting each year's diameter increment. Height growth was estimated by using height–diameter functions to predict the past height of a tree based on its past diameter.

(iii) Modelling individual tree biomass growth: we modelled the biomass growth of each species as a function of tree size, competition for light, species richness and a random plot effect: 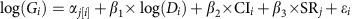, where *G*_*i*_, *D*_*i*_ and CI_*i*_ are, respectively, the biomass growth, stem diameter and crown illumination index of tree *i* growing in plot *j*; SR_*j*_ is the species richness of plot *j*; *α*_*j*_ is a species' intrinsic growth rate for a tree growing in plot *j*; *β*_1–3_ are, respectively, a species' growth response to size, light availability and species richness; and *ɛ*_*i*_ is the residual error. The structure of the growth model is adapted from Jucker *et al*.^27^ and was fitted using the *lmer* function in R. Model robustness was assessed both visually, by comparing plots of predicted versus observed growth, and through a combination of model selection and goodness-of-fit tests (AIC model comparison and R^2^). Across all species, individual growth models explained much of the variation in growth among trees^27^.

(iv) Scaling up to plot-level AWP: to quantify AWP at the plot level, we used the fitted growth models to estimate the biomass growth of all trees that had not been cored. For each plot, we then summed the biomass growth of all standing trees to obtain an estimate of AWP. Growth estimates were generated using the predict.lmer function in R.

### Tree regeneration

Field sampling to quantify regeneration was carried out in 2012, from late April to late August, following a sequential scheme starting in southern Europe (Mediterranean forest, Spain) to the latest one in northern Europe (Boreal forest, Finland), to ensure that all tree species had fully expanded leaves at the sampling time. Regeneration was quantified in a subplot located at the centre of each main plot, whose size varied according to number of juveniles found for each species: (i) 4 × 4 m (16 subplots of 1 × 1 m were taken when <10 individuals for each species were found within the 16 subplots) and reduced to (ii) 2 × 2 m (4 subplots of 1 × 1 m when >10 individuals for each species were found in these subplots). In each regeneration plot, we identified juveniles to species (individuals of at least 1 year old till 1.60 m high) of all focal tree species in each forest and recorded the abundance for each one. To standardize the sampling size to get a meaningful comparison in the abundance regeneration data among plots, we extrapolated the number of juveniles from 2 × 2 to 4 × 4 m, when necessary. Analyses were performed using a log10 transformation of the abundance data.

### Fine root biomass

On each plot for determining fine root biomass nine soil samples were taken from a predefined grid. The sampling was done in six countries during May–October 2012. The forest floor was sampled using a wooden frame of size 25 × 25 cm, and thereafter the mineral soil was sampled using a cylindrical metal corer with 36 mm of inside diameter. The mineral soil was sampled down to 20 cm, except for the plots in Poland (down to 40 cm) and in Spain (down to 10 cm). Samples were pooled by layer and plot into one sample. Living fine roots (diameter ≤2 mm) were separated from the soil samples by hand to two categories, tree roots and ground vegetation roots. After separation, the roots were washed with water to remove adhering soil. Subsequently, the roots were dried at 40 °C until constant mass and weighed for biomass. The root biomass was corrected with a correction factor for soil stoniness (CF_stones_=(100−(% stones)) × 100^−1^), where the respective volumetric stoniness was estimated with the metal rod method^37^,^38^ on each plot. For this study total tree fine root biomass for each plot was calculated (g m^2^) for the sampled soil layer (forest floor + sampled mineral soil).

### Wood decomposition

Flat wooden sticks (wooden tongue depressors made of *Betula pendula* wood) were placed to decompose at each plot of the exploratory platform. Each wooden stick was initially weighed (average of 2.5 g). As the weighing was done on air dry sticks, subsamples were weighed, dried at 65 °C for 48 h and reweighed to get a 65 °C dry mass correction factor.

Within each plot, three wooden sticks were placed on the bare soil after the natural litter layer had been locally removed, and fixed to the soil by placing chicken wire on top of it. The wooden sticks stayed in the field for different durations among regions (Supplementary Table 6) depending on the mass loss of the region's fastest decomposing litter species (target of 50–60 % mass remaining), that was placed in the field at the same time as the wooden sticks.

After field exposure wooden sticks were harvested, dried at 65 °C, and weighed. Mass loss of wooden sticks was expressed as the percentage of initial mass lost, calculated as followed: mass loss=100 × (initial mass−final mass) × initial mass^−1^.

### Litter decomposition

#### Litter collection and litterbag construction

Leaf litter from all target tree species of the cross-region exploratory platform was collected at tree species-specific peak leaf litter fall between October 2011 and November 2012. Except for the Finnish forests, where freshly fallen leaf litter was collected from the forest floor, litter was collected using suspended litter traps, which were regularly harvested at 1–2 week intervals. In all cases, litter was collected nearby, but not within the experimental plots. Litter was then air-dried and stored until the preparation of the litterbags.

Litterbags (15 × 15 cm) were constructed using polyethylene fabrics of two different mesh sizes. For the bottom side of the litterbags we used a small mesh width of 0.5 × 0.5 mm to minimize losses of litter fragments, while for the upper side we used a large mesh width of 5 × 8 mm to allow soil macrofauna access to the litter within bags. Litterbags were filled with 10 g of litter. For litter mixtures, litterbags were filled with equivalent proportion of each litter species. Subsamples of all litter species were weighed, dried at 65 °C for 48 h and reweighed to get a 65 °C dry mass correction factor.

#### Litterbag incubation

Within each experimental plot, three litterbags with the plot-specific litter type (either single litter species or specific mixtures) were placed on bare soil after the natural litter layer had been removed, and fixed to the soil by placing chicken wire on top of it. The litterbags were removed from the field when 50–60% of the initial litter mass of the region's fastest decomposing species was remaining (evaluated with an extra set of litterbags that were harvested regularly). As a consequence, the duration of litter decomposition varied among regions (Supplementary Table 6). This procedure ensured that litter was sampled at similar decomposition stages across all sites, facilitating meaningful comparisons of litter diversity effects.

#### Litter processing

Harvested litterbags were sent to Montpellier where they were dried at 65 °C. Litter was cleaned of pieces of wood, stones or other foreign material that occasionally got into the litterbags. Litter was then weighed, ground to a particle size of 1 mm with a Cyclotec Sample Mill (Tecator, Höganäs, Sweden). To correct for potential soil contamination during decomposition in the field, we determined the ash content of initial and final litter material on all samples and expressed litter mass loss on ash-free litter mass.

Litter mass loss was expressed as the percentage of mass lost from each litterbag, calculated as followed: mass loss=100 × (initial (ash free) mass−final (ash free) mass) × initial (ash free) mass^−1^.

### Microbial biomass

For soil sampling, each of the 209 plots was divided into nine 10 × 10 m subplots. A soil sample was taken from 5 of the 9 subplots and mixed to obtain one representative composite sample from each plot. Forest floor and mineral soil horizons (0–5 cm) were sampled separately. Soils were sieved fresh (4 mm), stored at 4 °C and analysed within 2 weeks. Sampling was performed in spring 2012 in Italy, Germany and Finland, and in autumn 2012 in Poland, Romania and Spain. No forest floor was collected from the plots in Germany.

Soil microbial biomass C was determined by the chloroform fumigation extraction method (ISO 14240-2 (ref. 39)), of 10 and 15 g (organic and mineral soil, respectively) soil, followed by 0.5 M K_2_SO_4_ extraction of both fumigated and unfumigated soils (soil:solution ratio, 1:5). Fumigations were carried out for 3 days in vacuum desiccators with alcohol-free chloroform. Extracts were filtered (Whatman no. 42), and dissolved organic carbon in fumigated and unfumigated extracts was measured with a Total Organic Carbon analyser (Labtoc, Pollution and Process Monitoring Limited, UK). Soil microbial biomass C was calculated by dividing the difference of total extract between fumigated and unfumigated samples with a *k*_EC_ (extractable part of microbial biomass C after fumigation) of 0.45 for biomass C^40^.

### Soil carbon stock

Soil sampling was carried out from May 2012 to October 2012 (that is, Poland in May 2012, Spain in June 2012, Finland and Germany in August 2012, Romania in September 2012 and Italy in October 2012). Nine forest floor samples and nine cores of mineral soil were collected from each plot and these were subsequently pooled into one sample per plot by each soil layer, that is, forest floor, 0–10 cm and 10–20 cm depths for samples from Germany, Finland, Italy and Romania. For Poland, the fixed depth was extended to 20–30 cm and 30–40 cm whereas for Spain it was only possible to sample up to the 0–10 cm layer due to the stoniness of the site. We oven-dried the samples at 55 °C to constant weight, sorted out stones and other materials, ground the forest floor first with a heavy-duty SM 2000-Retsch cutting mill, and we then took subsamples and ground it further into finer particles with a planetary ball mill (PM 400-Retsch) for 6 min at 280 r.p.m. The mineral soil samples were sieved through 2 mm diameter mesh. We carried out carbonate removal treatments for those soil samples whose pH value exceeded the threshold point and proved presence of carbonates when tested with a 4 N HCl fizz test. We used 6% (w/v) H_2_SO_3_ solution and followed the carbonate removal procedure described by Skjemstad and Baldock^41^. We took subsamples and further ground it into finer particles with a planetary ball mill (PM 400-Retsch) for 6 min at 280 r.p.m. before analysing soil organic carbon (SOC) with a Thermo Scientific FLASH 2000 soil CN analyser, Italy while following the dry combustion method^42^. Soil organic C stocks were estimated by multiplying the SOC concentrations with soil bulk density, relative root volume and relative stone volume^43^. We also determined the moisture content of the soil samples by oven-dried subsamples at 105 °C and the reported SOC stock is thus on 105 °C dry weight basis.

### Resistance to drought

For each plot, we randomly selected six trees among the 12 largest ones (that is, largest diameter at breast height, DBH). For the mixed plots, three trees per species were randomly selected among the six largest trees of each species. This selection was conducted as to only select dominant and/or co-dominant trees to avoid confounding factors related to light interception. From each selected tree, a wood core was extracted at breast height during the summers of 2012 and 2013. For each site, we selected 2 years with contrasting climatic conditions during the growing season (dry versus wet year) during the 1997–2010 period (see ref. 31 for full details). Latewood samples from these 2 years were carefully extracted from each wood core. The latewood sections from a given year and a given species in a given plot were bulked and analysed for their carbon isotope composition (δ^13^C, ‰) with a mass spectrometer. By only selecting latewood sections, we characterized the functioning of the trees during the second part of the growing season and avoided potential effects related to the remobilization of stored carbohydrates from the previous growing season^44^ or to a favourable spring climate. Plot-level δ^13^C was calculated as the basal area weighted average value of species-level δ^13^C measurements. Soil drought exposure in each forest stand was calculated as the stand-level increase in carbon isotope composition of latewood from the wet to the dry year (Δδ^13^C_S_). For more details on resistance to drought measurements, we refer to ref. 31.

### Resistance to insect herbivory

As for fungal pathogens sampling, we estimated insect herbivory on six trees per species in monocultures and three trees per focal species in mixed forests. The herbivory assessment was done once, from late spring to early summer (see periods on fungal pathogens protocol). The insect herbivory protocol was derived from the ICP Forests manual^45^. It was adapted to be better account for total insect damage by observing the whole tree crown, instead of the ‘assessable crown' only. Damage on crown exposed to sunlight and in the shade were recorded separately, as foliar loss may be also due to competition for light or natural pruning in the shaded part, particularly in heliophilous tree species. We considered damage as leaf area loss or shoot mortality, that is, defoliation. To estimate herbivore impact, we compared the sampled trees with a ‘reference tree', that is, a healthy tree with intact foliage in its vicinity. Two variables were recorded on each sampled tree, using binoculars and seven percentage classes for all proportion variables: 0, 0.5–1, 1–12.5, 12.5–25, 25–50, 50–75 and > 75%. Using binoculars, we estimated the proportion of defoliation in the living crown (that is, the crown excluding the dead branches) in both parts of the crown (sunlight exposed *P*_DL_ and in the shade *P*_DS_) and put the estimates in one out of seven percentage classes: 0, 0.5–1, 1–12.5, 12.5–25, 25–50, 50–75 and >75% damage. The assessment was done from at least two sides of the crown to account for all damage. When a different score was attributed from different sides to a focal tree, the mean of damage class median was used. The total per cent of defoliation was the calculated as the natural logarithm of the sum of *P*_DL_ and *P*_DS_.

### Resistance to mammal browsing

All plots were sampled using four 5 × 5 m subplots located in the same areas of each plot (Supplementary Fig. 7). Within each of the four 5 × 5 m subplots each woody species individual was visually inspected for browsing damage (bitten twigs). When browsing was found, the species was recorded, an estimation of the percentage of twigs browsed (between a height of 0.5–2 m) was made (biomass removed), and the stem diameter (at the base) and upper and lower limits of browsing were recorded. With these data, a plot-level average of percentage of twigs browsed was calculated, and resistance to mammal browsing was defined as 100% of twigs browsed.

### Resistance to fungal pathogens

Fungal pathogen damage was assessed over a 2-week period at each plot during the growing period, over 2 years. Foliage was collected from Italy (June–July 2012), Germany (July 2012), Finland (August 2012), Spain (June 2013), Romania (July 2013) and Poland (July–August 2013).

In each plot, the six trees with the largest DBH per species were selected for trees within monoculture plots, and three trees with the largest DBH per species for trees within mixture plots. Foliage (leaves and shoots) samples were collected from branches from two levels of the tree canopy (25–60 leaves and 10 current-year shoots per branch) for each focal tree species. The number of leaves sampled from each focal tree and the number of plots within each tree species richness levels are enumerated in Supplementary Table 7. Visual assessments for fungal pathogen damages were conducted on fresh leaves within one day of sampling. Leaves and shoots were assessed for four classes of fungal damages: oak powdery mildew and leaf spots for the broadleaved tree species, and rust and needle cast for the conifer species. The number of leaves or shoots with the respective damages per tree was recorded, as well as the number of leaves and shoots free from fungal pathogen damage, that is, healthy foliage. To obtain a value of healthy foliage at the plot level, the sum of all healthy foliage for all trees within the plot was calculated and this was divided by the total number of foliage replicates to acquire a plot-level proportion of healthy foliage. All assessments were conducted by one person to avoid observer bias. For details on the sampling effort, we refer to Supplementary Table 7.

### Bird diversity

Bird communities were sampled by means of standardized point-counts with one visit per plot. Four trained observers recorded all birds heard and seen within the plot, except flyovers, during a 15-min period, using a distance-limited detection radius within 80 m from the observer. Bird counts were performed during the first 4 h after sunrise in the breeding season, on days without adverse weather conditions such as strong wind or heavy rain. Bird species diversity was defined as the Shannon–Wiener diversity of bird species recorded per forest plot. Bird counts were carried out in April–June 2012 in Finland, Germany and Italy and in April–June 2013 in Poland, Romania and Spain.

### Bat diversity

Bat communities were sampled during one night per plot, with an automatic bat recorder (Sound Meter SM2BAT, Wildlife acoustics) located at the centre of the plot. Detectors were calibrated to pick up all bat calls and were programmed to record from 1 h before sunset to 1 h after sunrise. Recordings were carried out only during nights without rain, with low wind speeds (<30 km h^−1^) and with an ambient temperature >10 °C. Bats were identified to the finest possible taxonomic level on the basis of their calls by the same trained operator using Sonochiro v3.2.3 and Batsound 4.1 softwares and updated species lists for each country. Identification to species level was achieved for about 80% of the calls, but two pairs of *Myotis* spp (*Myotis mystacinus* and *M. brandtii*; *M. myotis* and *M. blythii*) could not be fully separated. Bat species diversity was defined as the total number of species (or species pairs) of bats recorded per forest plot. Bat sampling was carried out in April–June 2012 in Finland, Germany and Italy and in May–July 2013 in Poland, Romania and Spain.

### Understorey plant diversity

Each plot was subdivided in nine 100 m^2^ subplots of which the southwest, central and northeast subplots were used for the understorey surveys from May to August 2012. In each of these three subplots, the percentage cover of each vascular plant species was estimated in a vegetation quadrat of 5 × 5 m. These data were used to calculate Shannon–Wiener diversity of understorey plants. Plot-level understorey diversity was calculated as the average Shannon–Wiener diversity of the associated subplots.

### Earthworm biomass

Earthworm sampling was carried out in spring 2012 in Italy, Germany and Finland, and in autumn 2012 in Poland, Romania and Spain. Plots were divided in nine 10 × 10 m subplots. 1 sample per plot was taken in the centre subplot. Sampling close to tree stems was avoided and whenever possible performed, in between multiple, different tree species. At each sampling point earthworms were sampled by means of a combined method. First litter was hand sorted over an area of 25 × 25 cm^2^. After litter removal over an enlarged area of 0.5 m^2^, ethological extraction using a mustard suspension was applied. Finally hand sorting of a soil sample of 25 × 25 cm^2^ and 20 cm depth was performed in the middle of the 0.5 m^2^ area. Earthworms were preserved in ethanol (70%) for 2 weeks, transferred to a 5% formaldehyde solution for fixation (until constant weight), after which they were transferred to ethanol (70%) again for further preservation and identification. All worms were individually weighed, including gut content, and identified to species level. Results per unit area of the three sampling techniques were summed to determine the total earthworm biomass per m^2^.

### Calculating multifunctionality

Before analyses, all individual EF variables were standardized by transformation as follows: 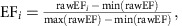 with EF indicating the final (transformed) ecosystem function value and raw EF indicating raw (untransformed) ecosystem function values. This way each transformed EF variable had a minimum of zero and a maximum of 1. Multifunctionality was then defined as the number of EFs in a plot that had a value above a threshold: 
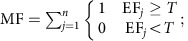
 where *n* is the number of EFs and *T* is the threshold value. Threshold values were defined as a certain integer percentage of maximum functioning^7^,^8^,^12^,^13^,^14^,^15^ within the country where the focal plot was located; multifunctionality was systematically calculated for all integer threshold levels from 1 to 99%; this is the most comprehensive approach to study ecosystem multifunctionality^12^. Although other studies defined ‘maxima' as slightly lower than actually observed maximum values^8^,^12^, we chose to explore expected diversity–multifunctionality relationships in the absence of complementarity and selection over an as large range of thresholds as theoretically possible, thereby demonstrating the symmetry of jack-of-all-trades effects (Figs 3 and 5), hence our choice for actually observed maxima. In plots with one (*n*=28), two (*n*=1) or three (*n*=1) missing EF values, we calculated the proportion of functions measured which exceeded the threshold multiplied with 16. Multifunctionality values are shown in Supplementary Data 1.

### Partitioning biodiversity effects

We developed a new approach that partitions net effects of biodiversity on individual functions into effects of complementarity and selection and that additionally partitions net effects of biodiversity on multifunctionality into effects of complementarity, selection and jack-of-all-trades mechanisms. While it was inspired by other approaches quantifying effects of complementarity and selection on diversity–functioning relationships^18^, our approach here is not mathematically equivalent, as we made a necessary adjustment to make it applicable to functions that are not measured at the species level within plots (for example, biogeochemical process rates).

We first calculated expected values for each single ecosystem function in (a) the absence of complementarity (EF_exp1_) and then (b) in the absence of both selection and complementarity (EF_exp2_), when assuming that effects of species on ecosystem functioning are proportional to their relative biomass^19^,^20^.


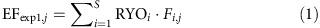



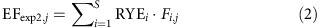


where *S* is the number of species present in the plot, RYO_*i*_ is the observed relative abundance (biomass) of species *i* in the mixture, that is, YO_*i*_ × *M*_*i*_^−1^, with YO_*i*_ being the observed biomass in mixture of species *i* and where *M*_*i*_ is the biomass value of a randomly selected monoculture plot of species *i* from the country in which the mixture is located. *F*_*i,j*_ is the value of function *j* and species *i* in the same randomly selected monoculture plot that was used as the input for *M*_*i*_. Exceptions were made for *Quercus robur*/*petraea* and *Betula sp.* observed in mixtures in Poland and for *Acer pseudoplatanus* observed in German mixtures: as these species did not occur in monocultures in their focal countries, *M*_*i*_ and *F*_*i,j*_ were respectively biomass and EF values from a randomly selected monoculture plot of species *i* from another country than the one from the focal plot. Finally, RYE_*i*_ is the expected relative abundance of species *i* in the mixture. RYE_*i*_ is defined as *S*^−1^, so that in the absence of selection, each constituent species of a mixed culture is expected to have equal abundance. Although true initial abundance is unknown in observational studies, plots were selected to be as even as possible in their species proportions (see above), so values of *S*^−1^ will minimize bias. Note that this partitioning approach was applied to other functions than standing biomass itself, to avoid circularity.

In addition, to estimate jack-of-all-trades effects, we calculated the expected function value if the mixture was replaced by a monoculture of a randomly selected species found in the mixture:


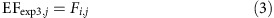


On average, EF_exp3*,j*_ values do not deviate from EF_exp2*,j*_ values, but averaging of function values of the species forming a mixture prevents EF_exp2*,j*_ values from being as extreme as EF_exp3*,j*_ monoculture values, which can cause positive biodiversity–multifunctionality relationships when multifunctionality is quantified as the number of functions exceeding moderate thresholds, but negative relationships at higher thresholds (Fig. 1). With these expected ecosystem function values we can calculate the effects of complementarity (EC) and selection (ES) on individual ecosystem functions as:


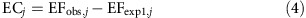



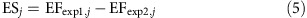


where EF_obs*,j*_ is the observed value of function *j*. Hence, our approach defines positive complementarity as the higher performance of a polyculture than would be expected from the monocultures from its constituent species and it defines positive selection as the dominance of high-performing species in polycultures. Expected and observed multifunctionality values (MF_obs_, MF_exp1_, MF_exp2_ and MF_exp3_) are then calculated as ref. 12:


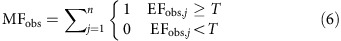



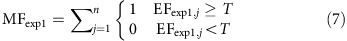



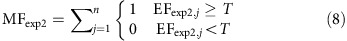



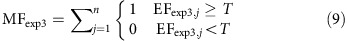


In this formulation functions contribute a value of one to multifunctionality if their value exceeds a threshold (*T*) and a value of zero when they do not. Observed and expected multifunctionality values can then be used to calculate the effects of complementarity (MEC), selection (MES) and jack-of-all-trades mechanisms (MEJ), as well as net effects of diversity, on multifunctionality (MEN):


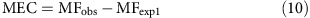



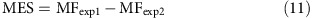



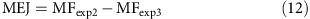



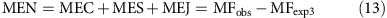


As partitioning biodiversity effects involved a randomization procedure (random selection of monoculture values), these effects were calculated 100 times: for each plot and each threshold value, final complementarity, selection and jack-of-all-trades effects equalled the average value of these 100 runs. For worked out examples of this approach, see Supplementary Figs 1 and 8.

To validate the ability of our approach to detect significant effects of complementarity or selection when observed relationships between diversity and ecosystem functioning are significant, we also applied our approach to an independent data set^46^. This confirmed that when relationships between diversity and functioning are significantly positive or negative (*P*<0.05), the effects of selection and complementarity are also significant and in the same direction (Supplementary Fig. 8).

### Testing for richness effects on ecosystem functioning

To analyse richness–multifunctionality relationships we first created full general linear mixed models (LMMs) for each (99 in total) threshold value, with multifunctionality as the response variable and seven potentially confounding factors as fixed effects/random effects. The fixed factors were: richness of target species, richness of non-target species, Pielou's species evenness, proportion of evergreen/coniferous trees, soil pH and altitude. Country (representing several climatic factors such as temperature and rainfall, as well as regional differences in species pools) and species composition (a factor listing all species present in the given plot) were included as random factors and a richness × country interaction effect was also calculated: we fitted random richness-slopes for each country. By including many potentially confounding variables in the LMMs, we greatly reduced the chances of detecting spurious diversity–multifunctionality relationships. As the proportion of evergreen species and coniferous species were highly collinear (only one non-conifer was evergreen, *Quercus ilex*), it would be problematic to put both as predictors in the same model. Therefore, for each threshold value of multifunctionality, we first compared models using Akaike Information Criterion (AIC) values and selected the model with the lowest AIC. LMMs with the proportion of coniferous trees were always (99 out of 99 tests) more parsimonious and therefore, in subsequent analyses, we did not include the proportion of evergreen trees as a covariate. We then tested whether fitting random slopes for species richness improved explanatory power, again using AIC to compare models. AIC values were lower for models without random slopes in 99 out of 99 tests, therefore random slopes were subsequently omitted from analyses. We then tested for significance of fixed effects using stepwise, backward model selection, in which we compared fuller models with more simple ones using likelihood ratio tests. We always retained species richness in the models, to derive effect sizes for all models, but we removed covariates which were non-significant in ≥80 out of 99 different models. By being conservative with omitting covariates, we reduced the chance of finding spurious richness–multifunctionality relationships whilst retaining the same model structure for all thresholds to allow comparison between them. Based on these criteria, altitude and proportion of coniferous trees were kept as covariates in the LMMs. We then investigated whether richness–covariate interaction effects or quadratic richness effects were significant using likelihood ratio tests for each threshold value. As all of these were non-significant in at least 82 (out of 99) cases, interaction and quadratic effects were not included in final LMMs. The final LMM structure, with altitude and the proportion of coniferous species as covariates and country and species composition as random factors was then used to investigate the effects of richness on multifunctionality, for all 99 threshold values^12^. In addition, we used the same model structure to investigate effects of richness on individual functions and their partitioned biodiversity effects and on multifunctional effects of complementarity, selection and jack-of-all-trades mechanism. We kept the model structure the same for all partitioned diversity effects and for both individual EFs and multifunctionality, so that we could compare the biodiversity effects of different mechanisms on different types of ecosystem functioning. To test whether relationships were consistent across countries, for country-based subsets of the data we also ran LMMs with the same structure (but without country as a random effect) to test for (partitioned) biodiversity effects on multifunctionality. Effect sizes for net richness effects or partitioned diversity effects were quantified and significance was assessed with likelihood ratio tests. We assumed a Gaussian error distribution for all models and checked whether this assumption was met (see respectively Supplementary Fig. 9 for the error distributions (approximating Gaussian distributions) and Supplementary Fig. 10 for the residuals versus fitted values (indicating homoscedasticity)). Model parameters were estimated using a Restricted Maximum Likelihood approach, while Maximum Likelihood estimates were used when comparing models with L-ratio tests.

To investigate whether relationships between diversity and multifunctionality were sensitive to the types of ecosystem functions considered in this study, we performed additional analyses in which multifunctionality was based on 15, rather than 16 functions. Hence, we performed 16 additional analyses, with in each analysis another of the 16 functions excluded from the multifunctionality metric. As in the main analyses, we ran LMMs where multifunctionality was predicted by species richness, altitude and the proportion of coniferous trees (fixed factors) and by country and species composition as random factors. We used a multiple threshold approach^12^ to investigate for each multifunctionality variable how the strength and significance of the diversity–multifunctionality relationship depended on the minimal threshold of functioning desired. Results are presented in Supplementary Fig. 2 and Supplementary Table 2.

### Biodiversity and multifunctionality in simulated communities

With the ‘mvrnorm' function in R 3.1.0, 15 artificial ecosystem functions were created, and each followed a standardized normal (*μ*=0, *σ*=1) distribution. This was repeated several times so that they varied in their degree of correlation with each other with correlation coefficients of −0.07; 0.00; 0.25; 0.50; 0.75 or 1.0 (respective *ϕ*_*x*_ values^47^: 0.00; 0.07; 0.30; 0.53; 0.77; 1.00). We simulated 20 values for each EF, corresponding to a regional pool of 20 species each with randomly assigned monoculture values of ecosystem functioning. Artificial communities (100) were then created with 1, 2, 3, 4 or 5 randomly selected species (each richness level was replicated 20 times) from the species pool. To eliminate potential selection effects (that is, species impacts on ecosystem functioning are not correlated with abundance), each species in a given community had the same abundance (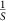, where *S* is species richness) in mixture. Ecosystem functioning (EF) was then calculated as: 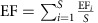, where *S* is the species richness *i* and EF_*i*_ is randomly assigned monoculture performance of species *i*. By randomly selecting species with equal abundance and by assuming that species effects on ecosystem functioning were additive, we created null expectations for diversity–functioning relationships in the absence of complementarity or selection effects. Multifunctionality was calculated in the same way as for multifunctionality of observed forest plots: 
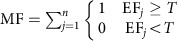
. For each threshold value of multifunctionality^12^, we ran LMMs with multifunctionality as the response variable and richness as the predictor. This procedure was repeated 100 times for each correlation coefficient between ecosystem functions, and average effect sizes of biodiversity were calculated for each threshold and correlation coefficient. The code of this simulation is available in Supplementary Data 1.

## Additional information

**How to cite this article:** van der Plas, F. *et al*. Jack-of-all-trades effects drive biodiversity–ecosystem multifunctionality relationships in European forests. *Nat. Commun.* 7:11109 doi: 10.1038/ncomms11109 (2016).

## Supplementary Material

Supplementary Information Supplementary Figures 1-10, Supplementary Tables 1-7 and Supplementary References.

Supplementary Data 1 R Code.
