# Supplementary material for: Jack-of-all-trades effects drive biodiversity–ecosystem multifunctionality relationships in European forests
Source: Nat Commun. 2016 Mar 24;7:11109. doi: 10.1038/ncomms11109 (PMC4820852; doi:10.1038/ncomms11109)
Supplement: Supplementary Information — Supplementary Figures 1-10, Supplementary Tables 1-7 and Supplementary References. [file ncomms11109-s1.pdf]

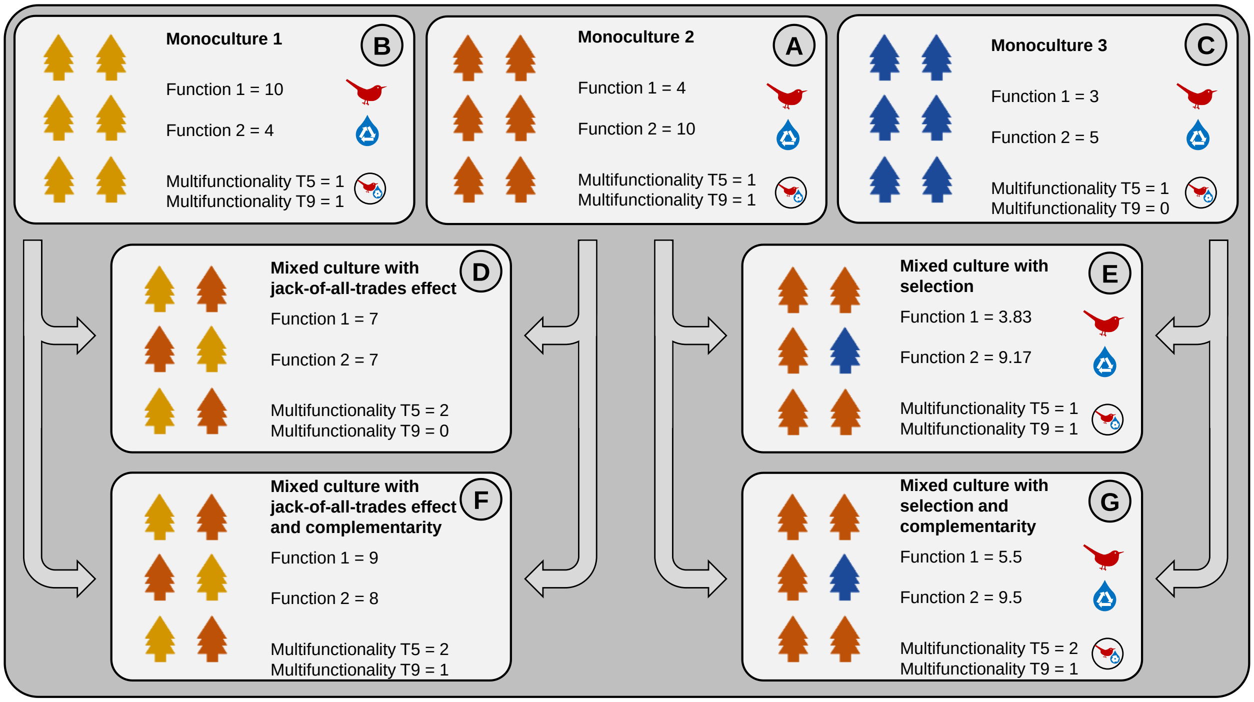

Supplementary Fig. 1. **Effects of selection, complementarity and jack-of-all-trades mechanisms can boost or decrease ecosystem functioning and multifunctionality in diverse communities.** A-C: Hypothetical monocultures with their values for two ecosystem functions and their values for multifunctionality based on a moderate threshold (T5) and a high threshold (T9). D,E: scenarios where function values in hypothetical mixtures of the monoculture species are derived additively from monoculture function values, leading to (D) a jack-of-all-trades effect or (E) a positive effect of selection. In D, function in the mixed culture can be derived from the weighted average of monoculture values: $EF_{exp1,j} = \sum_{i=1}^{S} RYO_i \cdot F_{i,j}$ (eqn 1 main article) = $EF_{exp2,j} = \sum_{i=1}^{S} RYE_i \cdot F_{i,j}$ (eqn 2 main article file; $EF_{exp1,j}$ and $EF_{exp2,j}$ are the same here because both species are equally abundant) = $0.5 \cdot 10 + 0.5 \cdot 4 = 7$ (function 1) and $0.5 \cdot 4 + 0.5 \cdot 10 = 7$ (function 2). Due to this additivity and the lack of abundance differences between component species, effects of selection and complementarity on individual functions and multifunctionality are 0. However, multifunctionality values in this mixture are either higher (for the moderate threshold level of 5) or lower (for the high threshold level of 9) than in the monocultures of component species, causing a positive jack-of-all-trades effect at a moderate threshold value, and a negative jack-of-all-trades effect at a high threshold value. In E, $EF_{exp1,j}$ $(= \sum_{i=1}^{S} RYO_i \cdot F_{i,j}$ $= 3.83$ (function 1) or 9.17 (function 2) is higher than $EF_{exp1,j}$ $(= \sum_{i=1}^{S} RYE_i \cdot F_{i,j}$ $= 3.5$ (function 1) or 7.5 (function 2) because the species with highest

monoculture values dominates. As a result, there are positive selection effects: $ES_j = EF_{exp1,j} - EF_{exp2,j}$ (eqn 5 main article file) = 0.33 (function 1) or 1.67 (function 2). F,G: scenarios where function values in hypothetical mixtures of the monoculture species are derived non-additively from monoculture function values. As a result, a combination of complementarity and jack-of-all-trades mechanisms (F) or complementarity and selection (G) alter functioning in diverse communities. In both examples, function levels are higher than expected based on additive effects (D,E). As a result, there are positive effects of complementarity: $EC_j = EF_{obs,j} - EF_{exp1,j}$ (eqn 4 main article file) = 2 and 1 (function 1 and 2 in F) or 0.67 and 0.33 (function 1 and 2 in G).

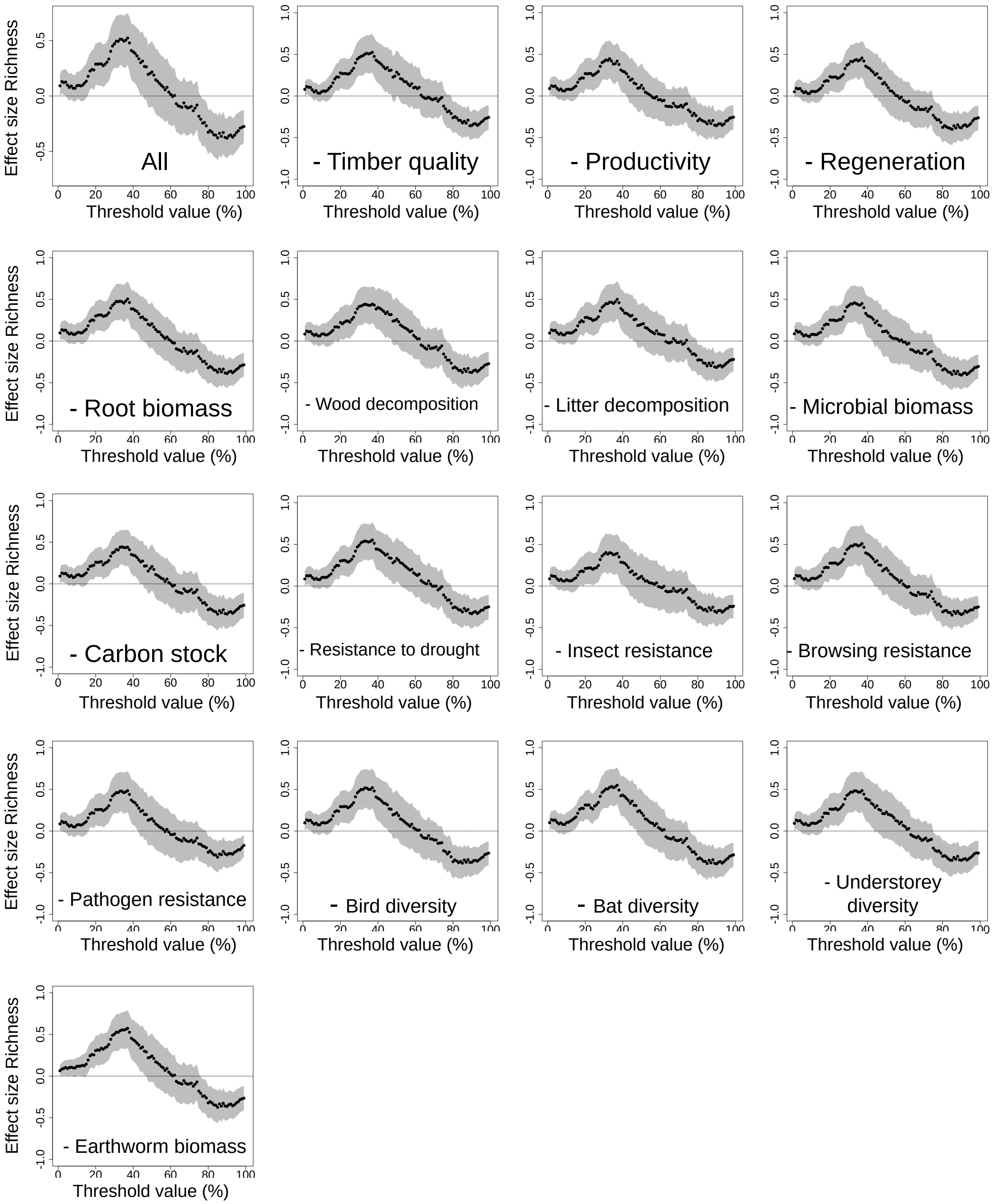

Supplementary Fig. 2. **Diversity-multifunctionality relationships are insensitive to the functions included.** Effect size (increase in number of functions > threshold per added species) by which species richness affects multifunctionality, both for overall multifunctionality and multifunctionality based on all but one discarded ecosystem function. Based on Linear Mixed Models (N = 209 plots). The grey polygons represent the 95% confidence intervals.

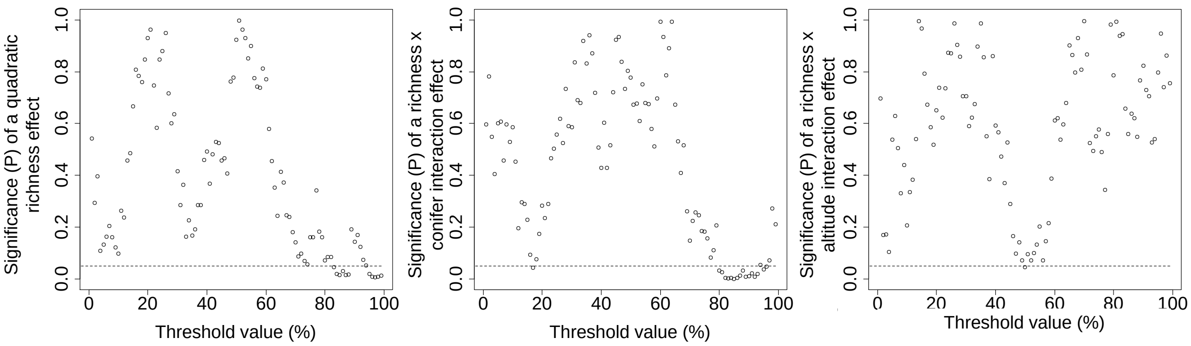

Supplementary Fig. 3. **The significance (*P* value) of a quadratic richness effect (left graph) a richness-conifer interaction effect (middle graph) and a richness-altitude interaction effect (right graph) on overall multifunctionality.** The quadratic richness effect was significant for 11 out of 99 tests, while the richness-conifer and the richness-altitude interaction effects were significant in 17 and 1 tests respectively. The horizontal, dotted line marks a significance level (*P*) of 0.05. Based on Linear Mixed Models (N = 209 plots).

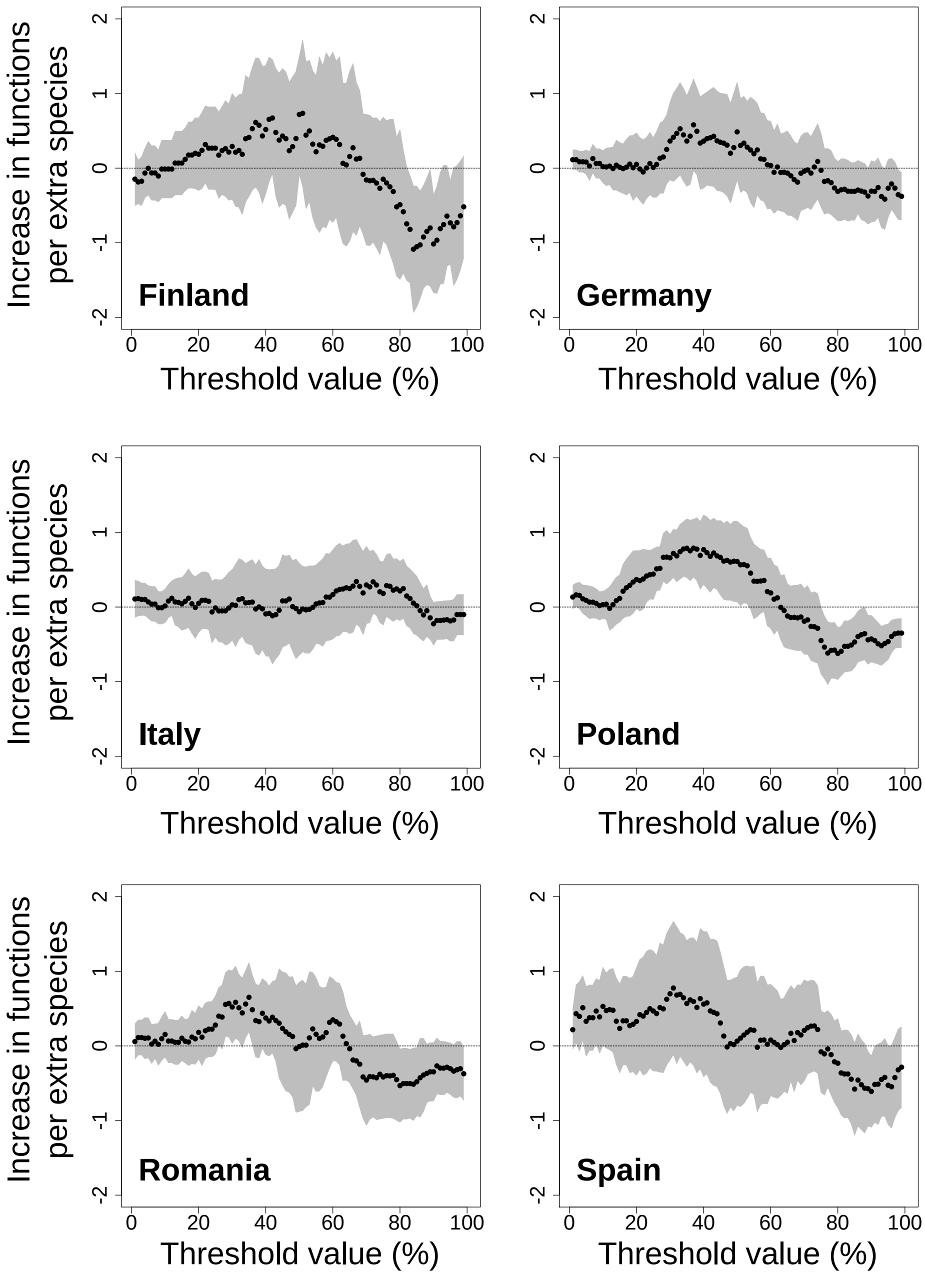

Supplementary Fig. 4. **The effect of tree biodiversity on ecosystem multifunctionality in different countries.** The biodiversity effect (change in total number of ecosystem functions per added species) is plotted as a function of the multifunctionality performance threshold value. The dotted, horizontal line represents a biodiversity effect of zero. Points above the horizontal line show positive effects of biodiversity, points below the line show negative effects. Based on Linear Mixed Models (N = 28, 38, 36, 43, 28 and 36 in respectively Finland, Germany, Italy, Poland, Romania and Spain).

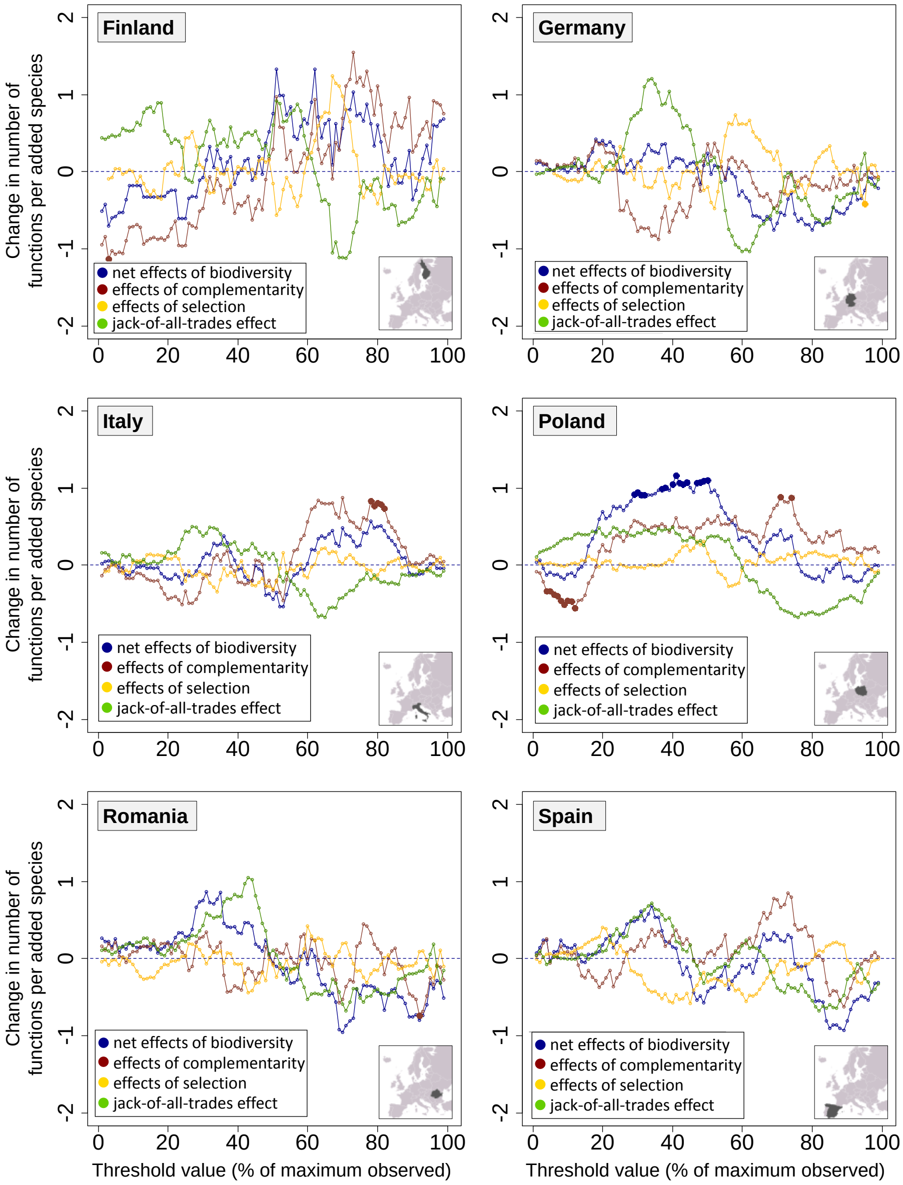

Supplementary Fig. 5. **The partitioned effects of forest biodiversity on ecosystem multifunctionality in different countries**. The biodiversity effects (change in total number of ecosystem functions per added species due to net, complementarity, selection or jack-of-all-trades effects) is plotted as a function of the multifunctionality performance threshold value. The dotted, horizontal line represents a biodiversity effect of zero. Points above the horizontal line show positive effects of biodiversity, points below the line show negative effects. Based on Linear Mixed Models (N = 28, 38, 36, 43, 28 and 36 in respectively Finland, Germany, Italy, Poland, Romania and Spain).

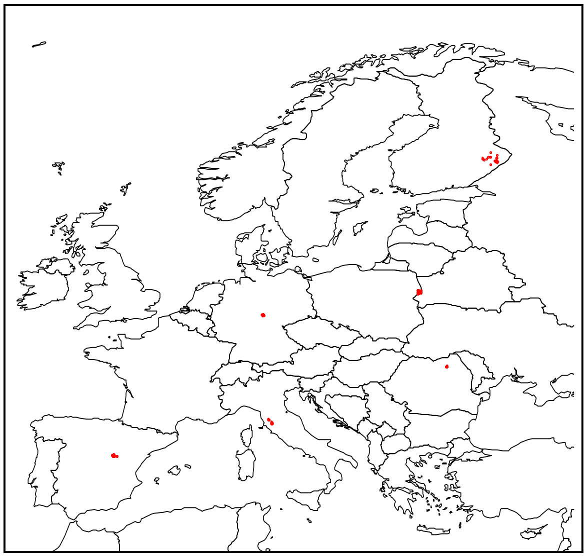

Supplementary Fig. 6. **Map of Europe with the locations of all plots.**

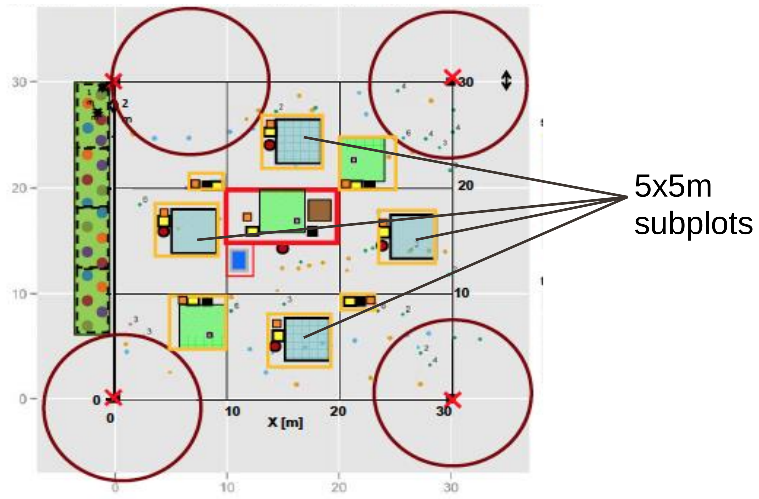

Supplementary Fig. 7. **Location of subplots where resistance to browsing was assessed.**

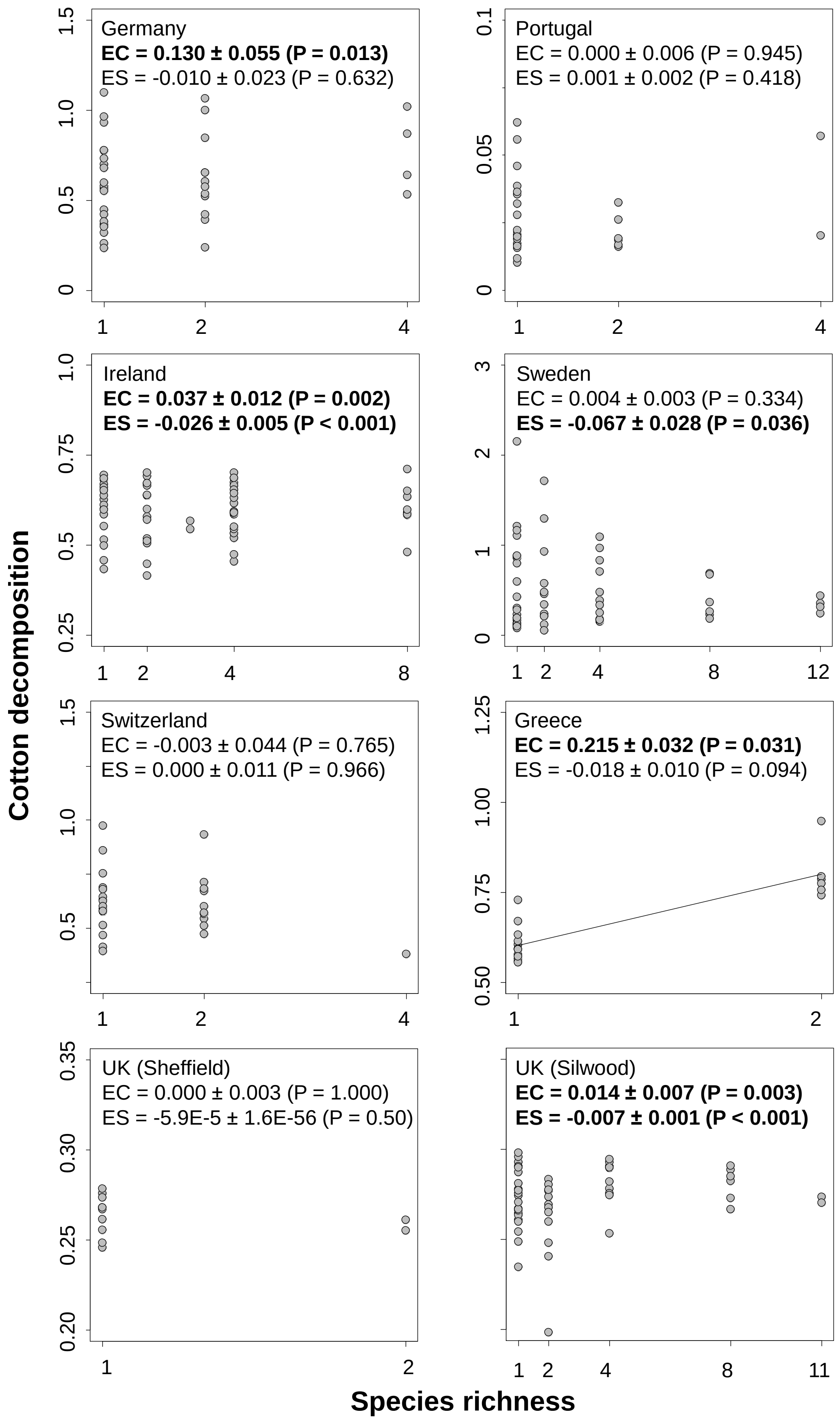

Germany
EC = 0.130 ± 0.055 (P = 0.013)
ES = -0.010 ± 0.023 (P = 0.632)
Portugal
EC = 0.000 ± 0.006 (P = 0.945)
ES = 0.001 ± 0.002 (P = 0.418)
Ireland
EC = 0.037 ± 0.012 (P = 0.002)
ES = -0.026 ± 0.005 (P < 0.001)
Sweden
EC = 0.004 ± 0.003 (P = 0.334)
ES = -0.067 ± 0.028 (P = 0.036)
Switzerland
EC = -0.003 ± 0.044 (P = 0.765)
ES = 0.000 ± 0.011 (P = 0.966)
Greece
EC = 0.215 ± 0.032 (P = 0.031)
ES = -0.018 ± 0.010 (P = 0.094)
UK (Sheffield)
EC = 0.000 ± 0.003 (P = 1.000)
ES = -5.9E-5 ± 1.6E-56 (P = 0.50)
UK (Silwood)
EC = 0.014 ± 0.007 (P = 0.003)
ES = -0.007 ± 0.001 (P < 0.001)
Cotton decomposition
Species richness

Supplementary Figure 8. **Biodiversity effects on cotton decomposition in 8 experiments are partitioning into complementarity and selection effects.** Observed relationships between biodiversity and cotton decomposition in 8 biodiversity experiments, as well as the average (± SEM) effects of complementarity (EC) and selection (ES) in mixed cultures, and their associated *P*-value, based on a Wilcoxon Signed Rank test. Decomposition data were acquired from Spehn *et al.* (*1*) and compositional data were acquired from Hector *et al.* (*2*). Only a subset of the plots (N = 297 in total: 36 in Germany, 26 in Portugal, 27 in Switzerland, 20 in Greece, 66 in Ireland, 58 in Sweden, 12 in Sheffield and 52 in Silwood), with (i) those monocultures of which all constituent species were also present in a monoculture and (ii) with non-missing decomposition or biomass data, was included. Our novel partitioning approach was used to estimate effects of complementarity and selection. A significant positive effect of diversity on decomposition was found in Greece, while positive trends were found in Germany, Ireland and Silwood and negative trends in Sweden. Significantly positive effects of complementarity in polycultures were found in Germany, Greece, Ireland and in Silwood, explaining the observed positive regiotionships / trends. Significantly (although weak) negative effects of selection in polycultures were found in Ireland, Sweden and Silwood.

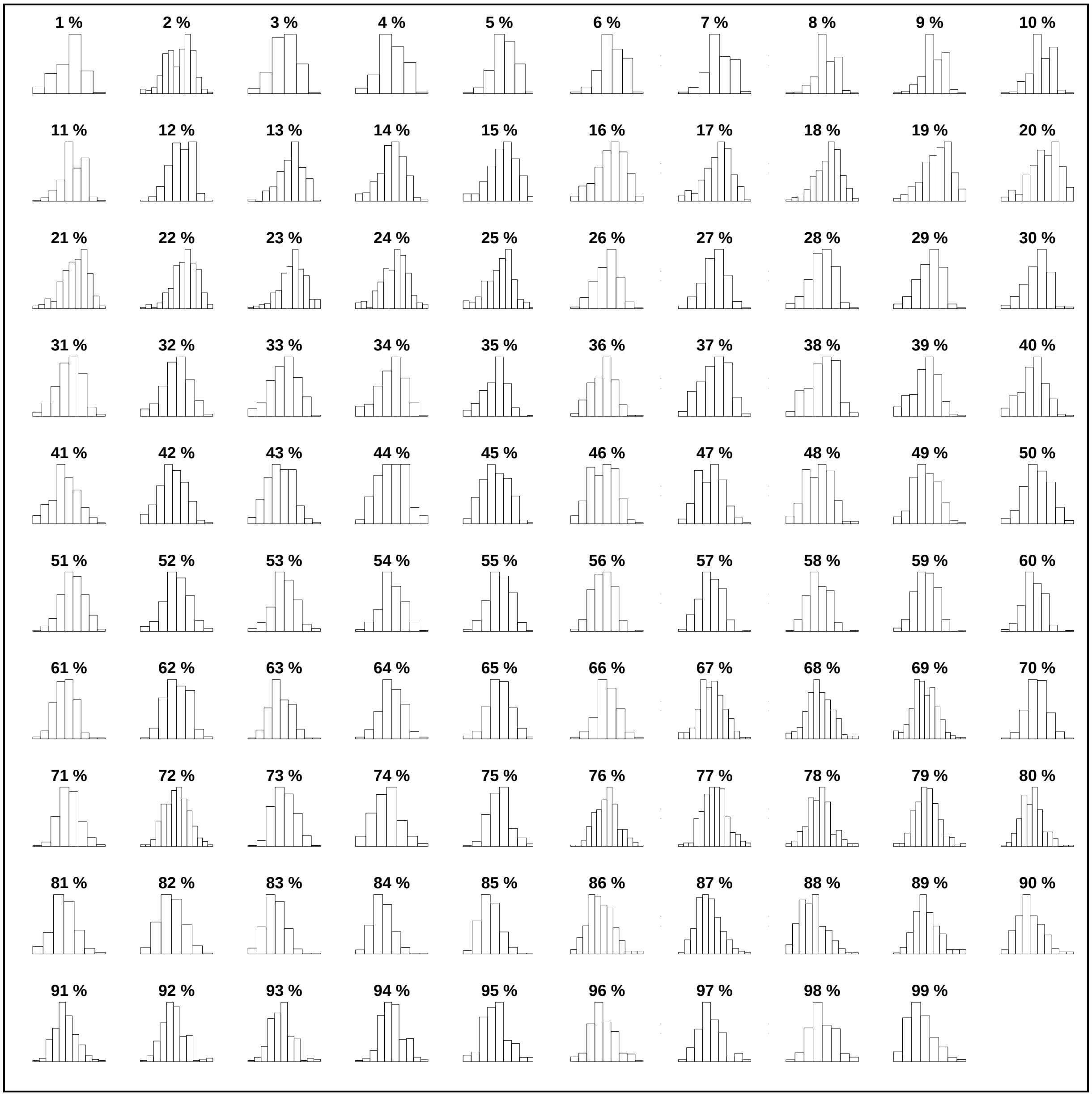

Supplementary Fig. 9. **Histograms of residual distributions of LMMs (N = 209) explaining overall multifunctionality, with different thresholds.**

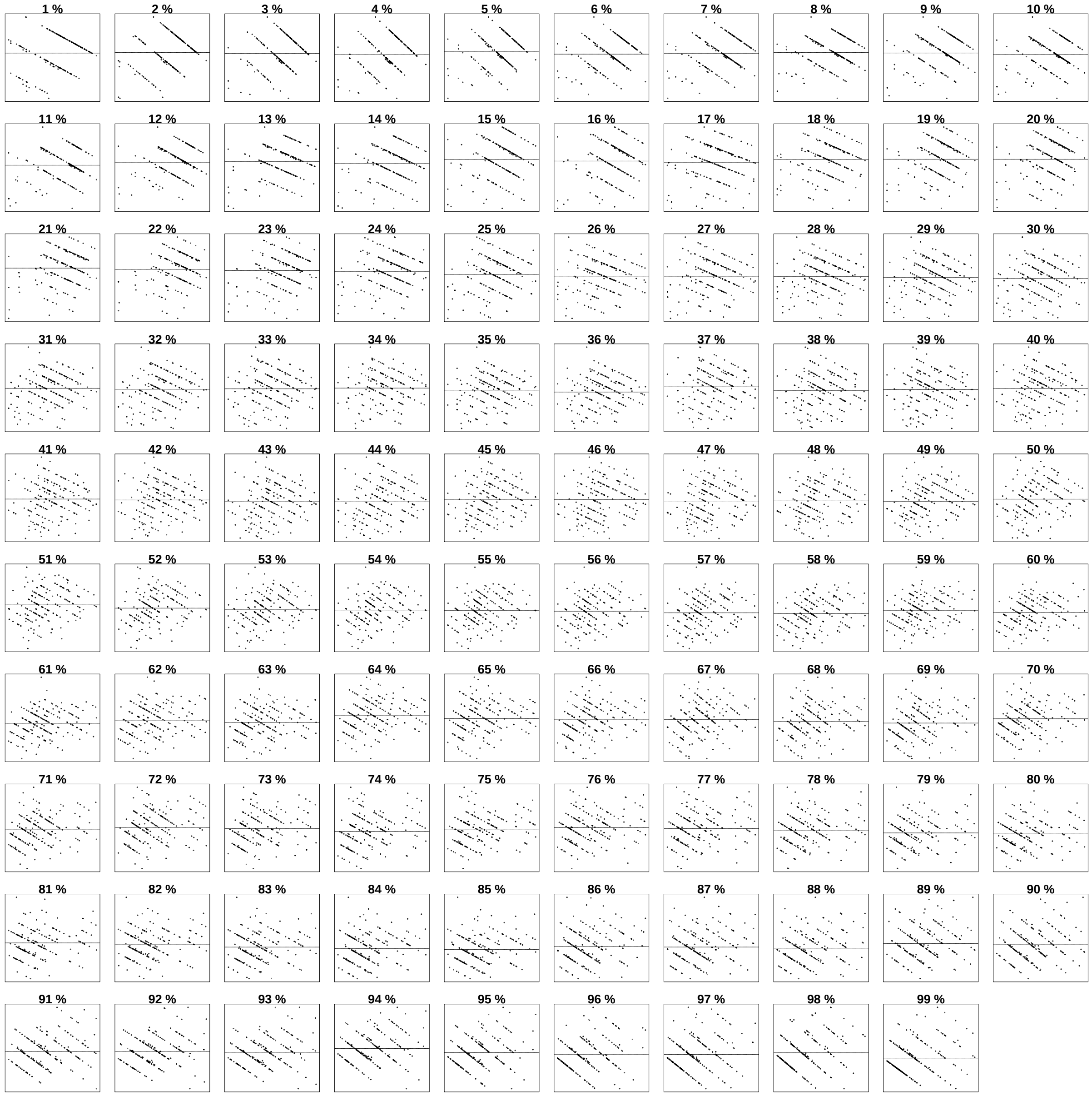

Supplementary Fig. 10. **Graphs showing the relationship between residual values of overall multifunctionality LMMs (N = 209) vs. fitted values, for all different threshold levels.**

Supplementary Table 1: **Summary statistics of the multiple threshold approach results.**

Scenario	Location	Positive biodiversity effects					Negative biodiversity effects				
		T_{min}	T_{max}	T_{mde}	R_{mde}	P_{mde}	T_{min}	T_{max}	T_{mde}	R_{mde}	P_{mde}
Overall multifuncionality	Europe	1%	45%	37%	0.52	13.0%	76%	99%	90%	-0.38	-9.5%
	Finland			51%	0.69	8.6%	82%	97%	90%	-1.07	-13.3%
	Germany	33%	38%	37%	0.80	15.0%	89%	99%	94%	-0.43	-8.1%
	Italy			67%	0.47	11.8%			90%	-0.24	-5.9%
	Poland	2%	48%	37%	0.77	19.2%	76%	99%	80%	-0.70	-17.4%
	Romania	26%	39%	35%	0.65	12.3%	99%	99%	82%	-0.46	-8.7%
	Spain	2%	10%	31%	0.78	14.6%	89%	95%	90%	-0.61	-11.5%
Multifunctional effects of complementarity	Europe	61%	99%	74%	0.43	13.3%	5%	28%	26%	-0.29	-9.0%
	Finland			73%	1.55	35.7%	3%	3%	3%	-1.13	-26.1%
	Germany			18%	0.39	9.06%			36%	-0.88	-20.2%
	Italy	78%	82%	70%	0.87	20.1%	20%	24%	24%	-0.51	-11.7%
	Poland	71%	74%	71%	0.88	20.4%	4%	12%	12%	-0.56	-12.8%
	Romania			76%	0.44	10.2%	92%	92%	92%	-0.73	-17.0%
	Spain			73%	0.85	19.5%			89%	-0.63	-14.5%
Multifunctional effects of selection	Europe	70%	86%	65%	0.14	4.4%	32%	33%	33%	-0.16	-4.9%
	Finland			67%	1.24	28.6%			51%	-0.56	-12.9%
	Germany			58%	0.73	16.9%	95%	95%	52%	-0.47	-10.9%
	Italy			65%	0.22	5.0%			34%	-0.34	-7.9%
	Poland			47%	0.31	7.05%			56%	-0.28	-6.4%
	Romania			60%	0.42	9.7%			43%	-0.44	-10.2%
	Spain			20%	0.41	9.3%			45%	-0.58	-13.4%
Multifunctional jack-of-all-trades effects	Europe	1%	51%	33%	0.68	20.9%	61%	99%	82%	-0.73	-22.4%
	Finland			51%	0.92	31.2%			71%	-1.11	-25.7%
	Germany			34%	1.21	27.8%			60%	-1.04	-23.9%
	Italy			27%	0.50	11.5%			65%	-0.67	-15.5%
	Poland			23%	0.49	11.3%			76%	-0.68	-15.7%
	Romania			43%	1.05	24.2%			71%	-0.68	-15.6%
	Spain			34%	0.71	16.5%			82%	-0.68	-15.6%

Values for indices generated by multiple threshold approach (*3*), with indices partitioned into effects of multifunctional complementarity, selection and jack-of-all-trades mechanisms. Analyses were conducted using both the complete dataset (all countries combined) and for countries separately. T_{min} / T_{max}: minimum / maximum threshold values where a significant positive or negative effect of biodiversity was found. T_{mde}: threshold of maximum (either positive or negative) biodiversity effect. R_{mde}: the strength of the biodiversity effect (change in number of ecosystem functions > threshold per added species). P_{mde}: Percentage of maximum possible diversity effect, i.e. R_{mde} divided by the maximum richness minus the minimum richness (*3*).

Supplementary Table 2. **Summary statistics of sensitivity analyses, where diversity-multifunctionality relationships were calculated for 15 (instead of 16) functions only.**

Missing function	Positive biodiversity effects						Negative biodiversity effects				
	T_{min}	T_{max}	T_{mde}	R_{mde}	P_{mde}		T_{min}	T_{max}	T_{mde}	R_{mde}	P_{mde}
none	1%	45%	37%	0.52	13.0%		76%	99%	90%	-0.38	-9.5%
Timber quality	2%	50%	37%	0.52	14.0%		79%	99%	89%	-0.36	-9.5%
Timber production	2%	42%	33%	0.45	11.9%		77%	99%	90%	-0.36	-9.5%
Tree regeneration	2%	43%	37%	0.46	12.2%		75%	99%	85%	-0.40	-10.6%
Root biomass	1%	45%	37%	0.50	13.4%		76%	99%	90%	-0.39	-10.4%
Wood decomposition	2%	47%	37%	0.44	11.8%		77%	99%	90%	-0.38	-10.0%
Litter decomposition	1%	45%	37%	0.50	13.3%		80%	99%	90%	-0.32	-8.5%
Microbial biomass	2%	42%	33%	0.46	12.2%		75%	99%	90%	-0.41	-10.9%
Soil carbon stock	1%	45%	34%	0.44	11.8%		77%	99%	85%	-0.36	-9.7%
Resistance to drought	2%	52%	37%	0.55	14.7%		79%	99%	90%	-0.33	-8.9%
Lack of insect herbivory	2%	41%	31%	0.40	10.7%		79%	99%	90%	-0.32	-8.5%
Lack of mammal browsing	1%	45%	37%	0.51	13.6%		77%	99%	85%	-0.35	-9.3%
Lack of pathogen damage	2%	42%	37%	0.48	12.9%		79%	99%	85%	-0.31	-8.3%
Bird diversity	1%	45%	37%	0.52	13.9%		75%	99%	85%	-0.38	-10.3%
Bat diversity	1%	47%	37%	0.55	14.6%		76%	99%	90%	-0.39	-10.5%
Understorey plant diversity	1%	45%	37%	0.49	12.9%		75%	99%	90%	-0.35	-9.4%
Earthworm biomass	2%	47%	37%	0.57	15.3%		76%	99%	85%	-0.37	-10.0%

Values for indices generated by multiple threshold approach (*3*) applied to overall multifunctionality (missing function = none) and multifunctionality based on all but one missing ecosystem function. T_{min} / T_{max}: minimum / maximum threshold values were a significant positive or negative effect of biodiversity was found. T_{mde}: threshold of maximum (either positive or negative) biodiversity effect. R_{mde}: the strength of the biodiversity effect (change in number of ecosystem functions > threshold per added species). P_{mde}: Percentage of maximum possible diversity effect, i.e. R_{mde} divided by the maximum richness minus the minimum richness, see Byrnes et al. 2014 (*3*).

Supplementary Table 3: **Species-level correlation coefficients between Ecosystem Functions.**

	TQ	TP	TR	RB	WD	LD	MB	CS	RD	IH	B	PD	BD	BaD	PD	EB
Timber quality (TQ)	1.00															
Timber production (TP)	0.28	1.00														
Tree regeneration (TR)	0.27	0.25	1.00													
Root biomass (RB)	**0.54**	**0.42**	0.11	1.00												
Wood decomposition (WD)	**-0.57**	-0.27	-0.30	-0.34	1.00											
Litter Decomposition (LD)	0.15	-0.31	**-0.49**	**0.45**	-0.17	1.00										
Microbial biomass (MB)	-0.13	-0.01	**0.53**	-0.35	0.06	**-0.41**	1.00									
Soil carbon stock (CS)	**0.53**	-0.32	0.14	-0.14	-0.23	0.18	0.29	1.00								
Resistance to Drought (RD)	**0.43**	0.35	**0.54**	**0.55**	**-0.62**	0.21	0.09	0.09	1.00							
Lack of insect herbivory (IH)	0.21	**0.41**	0.36	-0.33	-0.11	**-0.45**	0.35	0.29	0.11	1.00						
Lack of mammal browsing (B)	0.11	-0.17	-0.15	**-0.44**	-0.22	0.05	-0.09	**0.44**	-0.11	**0.44**	1.00					
Lack of pathogen damage (PD)	0.04	0.16	**0.57**	0.23	-0.32	-0.05	**0.49**	-0.07	**0.50**	0.01	-0.28	1.00				
Bird diversity (BD)	-0.17	**-0.42**	0.03	-0.36	**0.45**	-0.10	**0.43**	**0.45**	-0.4	-0.04	-0.12	-0.33	1.00			
Bat Diversity (BaD)	-0.09	-0.03	**-0.48**	0.19	0.03	**0.52**	-0.22	0.00	0.01	-0.31	0.19	0.04	-0.20	1.00		
Understorey plant diversity (PD)	0.19	0.01	-0.13	0.16	-0.28	**0.44**	-0.17	0.13	0.23	-0.03	0.21	0.05	-0.14	**0.67**	1.00	
Eartworm biomass (EB)	0.05	-0.02	**-0.59**	0.40	0.05	**0.62**	**-0.86**	-0.22	0.00	**-0.41**	0.08	-0.35	-0.29	0.39	0.27	1.00

Species-level correlation coefficients between Ecosystem Functions are based on average monoculture ecosystem function values, after correcting for country differences in functions. Correcting for country differences in functions was done by calculating residuals (average species function value – average country function value). Strong correlation coefficients between different variables (absolute coefficient coefficient value larger than 0.4) are shown in bold.

Supplementary Table 4. **Diversity-multifunctionality relationships in artificial communities.**

$\bar{r}$	φ_x	Positive biodiversity effects						Negative biodiversity effects				
		T_{min}	T_{max}	T_{mde}	R_{mde}	P_{mde}		T_{min}	T_{max}	T_{mde}	R_{mde}	P_{mde}
-0.07	0.00	1%	43%	29%	0.68	18.2%		56%	99%	70%	-0.67	-18.0%
0.00	0.07	1%	41%	30%	0.67	17.8%		58%	99%	72%	-0.67	-17.8%
0.25	0.30	1%	37%	28%	0.68	18.0%		63%	99%	70%	-0.68	-18.1%
0.50	0.53	1%	32%	30%	0.67	17.8%		67%	99%	73%	-0.67	-17.9%
0.75	0.77	1%	25%	29%	0.68	18.0%		73%	99%	72%	-0.67	-17.9%
1.00	1.00			31%	0.76	20.2%				72%	-0.76	-20.3%

Supplementary Table 5: **Quality class overview**

Quality class	Conifers	Broadleaved	Source
**A = 4**	one or more 5 m logs pruned or branch free; no curving; very few epicormics, very few to no pathologic defects; no waviness; no fissures/cracks	A: minimum of 5 m log almost branch free, very small and few epicormics or branches; no curving; no pathologic defects; no waviness; no fissures/cracks	(European Commission (*4-6)*; Mahler, Willmann & Wurster (*7*))
**B = 3**	one 5 m log almost branch free, no branches above 4 cm diameter above 5 m, few small epicormics; minor 1-sided curve acceptable, little taper; no mistletoe, minor pathologic defects; minor ovality; few branches	one or more 2 m logs largely branch free, no branches over 10 cm above 2 m, few small epicormics; 1-sided curve acceptable if otherwise acceptable; minor pathologic defects if wood damage is minimal; minor stem ovality; few branches	
**C = 2**	large branches along stem; curving, stem wounds, bumps, epicormics accepted	large branches along stem; curving, stem wounds, bumps, epicormics accepted	
**D = 1**	Stems are utilizable but are likely to yield less than 40 % usable timber	Stems are utilizable but are likely to yield less than 40 % usable timber	

Supplementary Table 6. **Duration of the litter and wooden sticks incubation per region**

**Region**	**Incubation start**	**Incubation end**	**Duration (days)**
Italy	06/06/2012	18/07/2013	407
Finland	20/06/2012	05/10/2013	471
Romania	16/07/2012	14/09/2013	362
Spain	03/09/2012	01/05/2014	605
Poland	18/09/2012	17/06/2013	269
Germany	08/10/2012	18/04/2013	190

Supplementary Table 7. **Details on sampling effort and characteristics of pathogen damage per species.**

Tree species	Country	Disease assessed	Plots with species	Total number of target trees	Number of plots, trees and leaves containing target species														
					1 species			2 species			3 species			4 species			5 species		
					Plot	Tree	Leaves	Plot	Tree	Leaves	Plot	Tree	Leaves	Plot	Tree	Leaves	Plot	Tree	Leaves
Castanea sativa	Italy	Leaf spots	17	56	2	12	699	4	12	690	5	14	810	5	15	913	1	3	180
Ostrya carpinifoila	Italy	Leaf spots	15	50	2	12	660	3	8	450	4	12	721	5	15	945	1	3	180
Quercus cerris	Italy	Leaf spots, Oak powdery mildew	18	59	2	12	720	4	12	686	5	14	820	6	18	1080	1	3	180
Quercus ilex	Italy	Leaf spots, Oak powdery mildew	20	66	2	12	675	4	12	661	6	18	1051	7	21	1261	1	3	180
Quercus petraea	Italy	Oak powdery mildew	17	53	2	12	660	3	9	450	5	15	870	5	14	881	1	3	180
Acer pseudoplatanus	Germany	Leaf spots	20	53	-	-	-	4	11	550	10	28	1400	6	14	725	-	-	-
Fagus sylvatica	Germany	Leaf spots	29	92	2	12	600	6	18	900	15	44	2200	6	18	900	-	-	-
Fraxinus excelsior	Germany	Leaf spots	23	72	1	6	300	6	18	900	12	37	1823	4	11	555	-	-	-
Quercus sp	Germany	Oak powdery mildew	16	45	1	6	305	2	6	300	8	22	1118	5	11	550	-	-	-
Picea abies	Germany	Rust, Needle cast	10	34	2	12	240	3	8	160	3	8	160	2	6	120	-	-	-
Betula pendula	Finland	Birch leaf spots	16	60	4	24	1200	8	24	1200	4	12	575	-	-	-	-	-	-
Picea abies	Finland	Rust, Needle cast	16	60	4	24	480	8	24	480	4	12	240	-	-	-	-	-	-
Pinus sylvestris	Finland	Rust, Needle cast	16	60	4	24	480	8	24	480	4	12	240	-	-	-	-	-	-
Quercus faginea	Spain	Leaf spots	22	75	4	21	1260	9	27	1620	6	18	1080	3	9	540	-	-	-
Quercus ilex	Spain	Leaf spots	15	51	3	15	900	6	18	1080	3	9	540	3	9	540	-	-	-
Pinus nigra	Spain	Rust, Needle cast	21	72	3	18	359	9	27	539	6	18	360	3	9	179	-	-	-
Pinus sylvestris	Spain	Rust, Needle cast	15	54	3	18	358	6	18	356	3	9	179	3	9	179	-	-	-
Acer pseudoplatanus	Romania	Leaf spots	12	42	2	12	671	3	9	540	4	12	676	3	9	506	-	-	-
Fagus sylvatica	Romania	Leaf spots	19	63	2	12	720	7	21	1260	7	21	1260	3	9	540	-	-	-
Abies alba	Romania	Rust, Needle cast	15	51	2	12	240	5	15	300	5	15	300	3	9	178	-	-	-
Picea abies	Romania	Rust, Needle cast	15	51	2	12	240	5	15	295	5	15	300	3	9	171	-	-	-
Pinus sylvestris	Poland	Rust, Needle cast	23	75	2	12	240	4	12	240	7	21	420	8	24	480	2	6	120
Betula pendula	Poland	Leaf spots	22	72	1	6	360	5	18	1080	6	18	1080	8	24	1440	2	6	360
Carpinus betulus	Poland	Leaf spots	25	81	2	12	720	6	18	1080	7	21	1260	8	24	1440	2	6	360
Quercus robur	Poland	Oak powdery mildew	23	75	1	6	360	3	12	720	9	27	1618	8	24	1440	2	6	360
Picea abies	Poland	Rust, Needle cast	23	75	2	12	220	5	15	300	6	18	360	8	24	480	2	6	120

SUPPLEMENTARY REFERENCES

1. Spehn, E. M. *et al*., *Ecol. Monogr*. **75**, 37-63 (2005).
2. Hector, A. *et al*., *Ecology* **95**, 2213-2220 (2010).
3. Byrnes, J. E. K. *et al*., *Methods Ecol. Evol.* **5**, 111-124 (2014).
4. European Commission. *EN 1316-1:1997: Hardwood Round Timber - Qualitative Classification - Part 1: Oak and Beech; German Version* (1997).
5. European Commission. *EN 1927-1:2008: Classification of Softwood Round Timber - Part 1: Spruces and Firs; German Version* (2008).
6. European Commission. *EN 1927-2:2008: Qualitative Classification of Softwood Round Timber - Part 2: Pines; German Version* (2008).
7. Mahler, G., Willmann, U. & Wurster, M. FVA - Forstliche Versuchs- und Forschungsanstalt Baden-Württemberg, http://www.fva-bw.de/forschung/wn/bwi_gueteanspr/arbeitsanweisung.pdf (2001).
